# Determinants of Health Care Technology Adoption Using an Integrated Unified Theory of Acceptance and Use of Technology and Task Technology Fit Model: Systematic Review and Meta-Analysis

**DOI:** 10.2196/64524

**Published:** 2025-12-30

**Authors:** Ayesha Thanthrige, Bruce Lu, Zaid Sako, Nilmini Wickramasinghe

**Affiliations:** 1 School of Computing, Engineering and Mathematical Sciences, La Trobe University, Plenty Road and Kingsbury Drive, Bundoora, Melbourne, Victoria, 3086, Australia, 610430601237; 2 School of Physics, Maths and Computing, Computer Science and Software Engineering, University of Western Australia, Perth, Australia

**Keywords:** unified theory of acceptance and use of technology, task-technology fit, health care technology adoption, SLR, UTAUT, TTF, meta-analysis, systematic literature review, PRISMA, Preferred Reporting Items for Systematic Reviews and Meta-Analyses

## Abstract

**Background:**

Health care technology adoption is key to improving patient care, enhancing operational efficiency, and ensuring better health outcomes. Examining the determinants that influence the acceptance and sustainable use of health care technologies is crucial for system developers, health care providers, and policymakers. The Unified Theory of Acceptance and Use of Technology (UTAUT) and task-technology fit (TTF) theoretical models offer a comprehensive framework to assess these determinants systematically, with UTAUT focusing on usage intentions (UI) and TTF emphasizing task-technology alignment for system usefulness, usability, and satisfaction.

**Objective:**

This systematic review and meta-analysis aimed to identify and analyze the key factors influencing the adoption of health care technologies based on an integrated UTAUT and TTF framework. By synthesizing existing literature, the study seeks to provide valuable insights for stakeholders to implement innovative and effective solutions in the health care domain.

**Methods:**

A search was conducted across a range of databases, including MEDLINE and Embase, IEEE Xplore, ScienceDirect, Scopus, CINAHL, Google Scholar, and Web of Science. Inclusion criteria covered studies applying either the UTAUT model, the TTF model, or both to health care technology adoption, published in English between 2012 and 2025. Exclusion criteria included nonquantitative studies, studies not focused on a health care setting, and those lacking sufficient data for meta-analysis. The reviewers collaborated to decide on the final papers for inclusion in the review through Covidence, the Cochrane Collaboration’s platform for systematic reviews. Data collection involved extracting quantitative data (eg, sample sizes, reliabilities, and standardized path coefficients) analyzed using meta-analytic techniques with a random-effects model in R software (R Development Core Team) to combine findings and calculate effect sizes.

**Results:**

A total of 50 studies [Bibr R1](35 UTAUT with 20,723 participants and 15 TTF with 4041 participants) met the inclusion criteria, representing various health care technologies, such as electronic health records, telemedicine platforms, and mobile health apps. The meta-analysis revealed that performance expectancy emerged as the most significant predictor of UI (β=.304; *P*<.001), while UI was the primary predictor of usage behavior (β=.199; *P*<.001). Other UTAUT predictors included effort expectancy (β=.177; *P*<.001), social influence (β=.167; *P*<.001), and facilitating conditions (β=.105; *P*<.001). For TTF, technology characteristics had the strongest effect on TTF (β=.445; *P*<.001), followed by TTF on UI (β=.271; *P*<.001) and task characteristics on TTF (β=.263; *P*<.001). Variability across settings and regions suggests contextual influences, with high heterogeneity (*I*²=81.90%‐94.87%).

**Conclusions:**

This study provides valuable insights for enhancing health care technology adoption by integrating UTAUT and TTF, highlighting performance expectancy, effort expectancy, social influence, facilitating conditions, task characteristics, technology characteristics, and TTF as key drivers. The findings, assessing system usefulness, usability, and satisfaction, can guide interventions to improve adoption and health care delivery.

## Introduction

### Background

The Unified Theory of Acceptance and Use of Technology (UTAUT) has emerged as a comprehensive framework to identify the factors influencing technology usage and acceptance. Originally, this model was proposed by Venkatesh et al [1], and UTAUT integrates constructs from 8 models, namely Technology Acceptance Model, Theory of Reasoned Action, Motivational Model, Theory of Planned Behavior, Combined Technology Acceptance Model and Theory of Planned Behavior, Model of Personal Computer Utilization, Innovation Diffusion Theory, and Social Cognitive Theory. The theory identifies four key constructs, namely performance expectancy (PE), effort expectancy (EE), social influence (SI), and facilitating conditions (FC) that directly influence usage intention (UI) and usage behavior (UB) [1]. Complementing UTAUT, the task-technology fit (TTF) model, introduced by Goodhue and Thompson [2], focuses on the alignment between task requirements (task characteristics [TC]) and technology functionalities (technology characteristics [TechC]) [2] to predict UI and technology use, particularly relevant for assessing system usefulness, usability, and satisfaction.

The health care sector is heavily dependent on technology for enhancing patient outcomes and operational efficiencies, which provides opportunities for the application of UTAUT and TTF. With the rapid digital transformation in health care, the adoption of technologies, such as telemedicine, mobile health (mHealth) apps, integrated technologies, such as electronic health records (EHRs), wearable devices, machine learning, artificial intelligence, and other health information systems, is important [3,4]. The application of UTAUT in this setting can provide insights into how clinicians and patients interact with these technologies and the aspects that can influence their acceptance of technologies. Similarly, TTF offers insights into how well these technologies align with health care–specific tasks, enhancing adoption through functional fit [5].

Previous studies have revealed that PE, the degree to which using a technology will provide advantages to the user in executing certain tasks, is a significant predictor of technology adoption in health care. For example, prior studies have indicated that health care professionals are more likely to adopt EHR systems if they believe these systems will enhance their job performance by improving patient care and increasing efficiency. Similarly, EE notably affects the UI of health technologies. Technologies that are identified as easy to use are more likely to be adopted by health care professionals and patients [6]. SI, defined as the degree to which persons observe others believe that they should use the new system. This factor also plays a critical role in technology adoption in health care. For instance, peer influence, recommendations from superiors, and health care leaders can significantly influence the acceptance and use of health technologies. This is particularly related in health care settings where teamwork and collaboration are important [7,8]. FC, which refers to the extent to which a user believes that organizational and technical infrastructure supports the use of the system, is also critical in the health care context. Technical support, sufficient resources, and adequate training programs can substantially influence the adoption of health technologies. Studies have shown that when health care professionals feel supported by their organization in terms of resources and necessary training, they are more likely to adopt and use new technologies [9,10]. Additionally, TTF research highlights that alignment between TC and TechC, as seen in studies like [11], enhances adoption by ensuring technologies meet specific task needs.

The application of UTAUT in the health care domain demands a detailed examination. This study extends this examination by integrating TTF to provide a more robust and holistic view of adoption determinants. This study aims to conduct a systematic literature review (SLR) and meta-analysis to synthesize this research on the application of UTAUT and TTF specifically in the health care context, bridging a critical gap in the literature. By analyzing studies that apply UTAUT and TTF to diverse health care technologies, this research seeks to identify the key elements influencing technology acceptance. A thorough review of the literature reveals that while UTAUT has been widely applied in various industries, there are still inconsistencies in the findings across different studies, even in the health care setting. For example, while some studies highlight PE as the most significant element of technology acceptance in health care [12,13], some studies emphasize that the role of SI or FC is significant [14,15]. However, some studies demonstrate that SI or FC do not significantly impact technology adoption [16,17]. These inconsistencies emphasize the requirement for a systematic review and meta-analysis to integrate the findings and provide a clear and solid understanding of the aspects influencing technology acceptance in health care. This study will use meta-analytic techniques to combine the findings from multiple studies gathered through SLR. Therefore, this study will increase statistical power, providing more reliable findings of the effect sizes of various UTAUT and TTF constructs. Multimedia Appendix 1 [11,12,15-62] summarizes previous UTAUT and TTF studies.

Moreover, UTAUT has been extended and modified in various studies to better fit the specific context of health care. For example, Cimperman et al [6] extended the UTAUT model to include perceived security as a factor impacting the adoption of health information technologies (HITs). Similarly, researchers incorporated additional constructs, such as anxiety, data security, motivation, and perceived benefits, to study the acceptance of telehealth equipment by patients and clinicians, providing a stronger understanding of the elements influencing technology adoption in health care [18-20]. Likewise, TTF has been adapted to assess performance impact, enriching the model’s applicability [21].

### Research Model and Hypotheses Development

This study specifically focuses on the health care setting, examining how PE, EE, SI, and FC influence UI and UB. This analysis is extended by integrating the TTF model to assess how TC and TechC influence TTF and subsequently UI. The research model suggests that PE (H1), EE (H2), SI (H3), and FC (H4) are the primary drivers of UI. Furthermore, FC (H5) and UI (H6) significantly influence UB. The TTF model posits that TC (H7) and TechC (H8) positively affect TTF, which in turn influences UI (H9), providing a comprehensive framework for evaluating system usefulness, usability, and satisfaction.

PE refers to the extent to which an individual perceives that using a specific technology will assist them in achieving gains in health management and outcomes [1]. Tian and Wu [22] found that PE is the most critical factor influencing the continuance intention of health care technology users, particularly among older patients with chronic diseases. Zhou et al [63] highlighted the importance of PE in driving user intentions by showing that the perceived usefulness of mHealth apps was a critical factor in their adoption. The perception that health care technologies can effectively manage health issues and improve health outcomes drives users’ intentions to adopt and continue using these technologies [22,23,64]. However, a study conducted by Arfi et al [24] revealed that PE has no impact on the intention to use the Internet of Things (IoT) for eHealth. Therefore, H1 was proposed: PE will be significantly and positively linked with UI within the health sector. EE is defined as “the degree of ease associated with the use of the system” [1]. Venugopal [25] found that EE positively impacts the UI of clinical staff toward the use of EHRs and telemedicine. Qvist et al [19] emphasized that ease of use significantly affects users’ satisfaction and their intent to continue using health care services. EE was found to positively influence both PE and UI, indicating that ease of use leads to higher adoption rates [26]. This relationship is consistent across various health technologies, including telemedicine and chronic disease management tools, highlighting the importance of user-friendly designs to enhance technology adoption and usage in health care [18]. In a comprehensive review of UTAUT applications, EE consistently emerged as a significant factor influencing the acceptance and usage of HITs [27]. For example, Lathifah et al [28] found that EE was an important element predicting the intention to adopt HIT among community health workers in Indonesia. However, a study conducted by Schmitz et al [29] identified EE as a nonsignificant factor impacting the UI to use virtual doctor appointments. Thus, H2 was formulated: EE will be significantly and positively associated with UI within the health sector. SI refers to the degree to which an individual perceives the importance of what others believe they should use a new system [1]. This hypothesis is supported by the recognition that social factors, such as friends’ recommendations, family, and health care providers, play an important role in shaping users’ intentions to adopt health care technologies. Numerous studies have shown that SI is a strong determinant of technology adoption among health care professionals [30,65]. For instance, research by Wu et al [62] found that SI significantly influences the intention to continue using health care technologies among older users. However, García de Blanes Sebastián et al [31] demonstrated that SI is a nonsignificant factor that impacts the adoption of virtual assistants. Additionally, SI affects consumers’ trust and information quality perception, which in turn influences their intention to use digital health care solutions during the COVID-19 pandemic [30,32,33]. Therefore, H3 was proposed: SI will be significantly and positively associated with UI within the health sector. FC refers to the extent to which an individual perceives that there is sufficient organizational and technical infrastructure to support the use of a system [1]. This includes the availability of resources, knowledge, and technical support necessary for the effective use of health care technologies. Venugopal [25] found that FC significantly influenced the intention to use EHR and telemedicine among clinical staff. Access to necessary technical support and infrastructure was crucial for adoption. Prior research has shown that FC significantly influences UI in health care [66]. For instance, Jewer [66] found that FC significantly influenced the intention to use the emergency department wait times website, while another study led by Araújo et al [34] showed that FC is a nonsignificant predictor of UI. Thus, H4 was suggested: FC will be positively and significantly associated with UI within the health sector. The empirical findings indicate that FC significantly impacts UB. Studies have shown that technical support and organizational resources facilitate the actual use of technologies in health care settings [67,68]. For instance, Cao et al [67] demonstrated that FC was closely associated with the UB of mHealth apps among users in Japan, and another study conducted by Owusu Kwateng et al [35] revealed the positive impact of FC on UB. Therefore, H5 was proposed: FC will be positively and significantly associated with UB within the health sector. UI is defined as the individual’s readiness to use the technology. In health care, UI is a strong predictor of actual technology use. Numerous studies have validated the significant relationship between UI and UB [62,69]. For example, Wu et al [62] found that the intention to use mHealth apps significantly predicted their actual usage among health care professionals in China. Thus, H6 was proposed: UI will be significantly and positively associated with UB within the health sector. TC refers to the specific tasks health care professionals and patients need to perform, such as clinical decision-making or patient monitoring, while TechC encompasses the features and functionalities of technologies designed to support these tasks. Wang et al [11] demonstrated that a strong TC-TechC alignment enhances TTF, which significantly predicts UI in wearable device adoption. However, Kang et al [36] found TC-TTF to be nonsignificant, suggesting context-dependent effects. Thus, the following hypotheses are proposed: (1) H7: TC will be positively associated with TTF, (2) H8: TechC will be positively associated with TTF, and (3) H9: TTF will be positively associated with UI.

**Figure 1.**
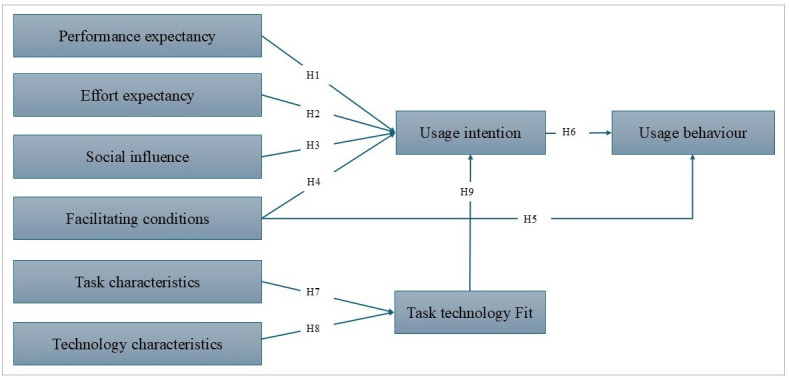
Integrated Unified Theory of Acceptance and Use of Technology and task-technology fit research model.

The research model (Figure 1) provides a comprehensive framework for examining the determinants influencing the adoption of HITs in health care settings. By examining the core constructs of UTAUT, this integrated UTAUT and TTF study aims to offer valuable insights into the determinants of technology acceptance among health care professionals.

## Methods

### Study Selection

To systematically review and meta-analyze the adoption of the integrated UTAUT and TTF models in health care settings, we followed a structured process based on the guidelines of the PRISMA (Preferred Reporting Items for Systematic Reviews and Meta-Analyses) and methodologies from prior meta-analytic studies [70]. Checklist 1 provides the PRISMA checklist. We conducted database searches on several databases, including MEDLINE and Embase, IEEE Xplore, Science Direct, Scopus, CINAHL, Google Scholar, and Web of Science, applying a combination of health care and adoption terms. Search results were imported into EndNote (Clarivate) to remove duplicates and then uploaded into Covidence software (Veritas Health Innovation Ltd). Multimedia Appendix 2 provides the MEDLINE search strategy, with additional database search strings.

### Eligibility Criteria

Studies that met the criteria listed in Textbox 1 were included to ensure the relevance and quality of the included studies.

Textbox 1. **Inclusion criteria.**

- The studies must focus on health care services and applications delivered via digital technologies, including electronic health records, mobile health, telehealth, and other health information technologies.
- The publications must be in English.
- The studies must discuss quantitative data in detail, for example, sample sizes and reliabilities (composite reliability or Cronbach α and standardized path coefficients [β]).
- Studies published between 2012‐2025.

The selection process involved a 2-step screening approach. First, titles and abstracts of the articles identified through the search were reviewed for relevance. All articles that satisfied the inclusion criteria were then subjected to a full-text review. Covidence was used as a tool for screening, full-text review, and management. Each title and abstract of the articles was assessed independently by 2 reviewers (AT and ZS) to minimize selection bias. Discrepancies between the reviewers were resolved through a third reviewer (NW). Articles that passed the initial screening were reviewed in full text independently by 2 reviewers (AT and ZS). Discrepancies of the full-text review between the reviewers were resolved through a third reviewer (NW).

### Coding Data

After gathering the related articles, the basic details were coded for each study. This included the title, publication year, author, journal, study design, main theory, and country. Quantitative data were gathered separately, focusing on the relationships between independent and dependent variables. These data included the size of the sample, reliability measures, composite reliability (CR), Cronbach α, validity (average variance extracted), standardized effect sizes (β-based), and significance of results (2-tailed *t* test and *P* values). To increase the number of included path coefficients and improve the precision of the meta-analysis, constructs with different labels but similar conceptual meanings and definitions to those in the UTAUT and TTF models were merged into a single factor [37,71]. For instance, behavioral intention is viewed as UI. As a result, 50 studies were considered for this meta-analysis.

### Statistical Analysis

A meta-analysis was performed to compile the 207 corrected estimates from 50 publications using R software. This method offers a consolidated perspective of research findings by quantitatively integrating both significant and nonsignificant results into combined outcomes [71]. Meta-analysis helps reinforce existing findings by consolidating results from multiple studies, thereby providing a more robust and reliable conclusion. Additionally, it identifies gaps in empirical evidence, highlighting areas where research is lacking or inconsistent, which in turn guides future research directions [14]. Furthermore, meta-analysis is useful for hypothesis testing, as it combines data from various sources to test the overall validity of research hypotheses [71]. In meta-analysis, 2 main statistical models are used to estimate the overall effect, namely the fixed-effect model and the random-effects model. Considering the included publications in this study, which come from various countries and report different effect sizes, we assumed that the random-effects model using R software would be more appropriate for calculating the weighted mean effect sizes for each UTAUT and TTF path relationship [72]. Effect sizes were computed as weighted mean path coefficients (β) using a random-effects model in R software, with weights based on inverse variance to account for variability in study designs, sample sizes, and health care contexts. This model was chosen to accommodate the diverse effect sizes and study origins, ensuring a generalizable summary effect. Heterogeneity was evaluated using *I*² metrics, with values ranging from 81.9% to 94.87% indicating substantial heterogeneity, likely due to regional, cultural, or technological differences across the 35 UTAUT and 15 TTF studies. Publication bias was not formally assessed; however, including both significant and nonsignificant results reduces potential bias. Future studies should use funnel plots and Egger test to evaluate publication bias explicitly. This approach ensures robust and generalizable findings across diverse health care settings.

## Results

### Overview

**Figure 2.**
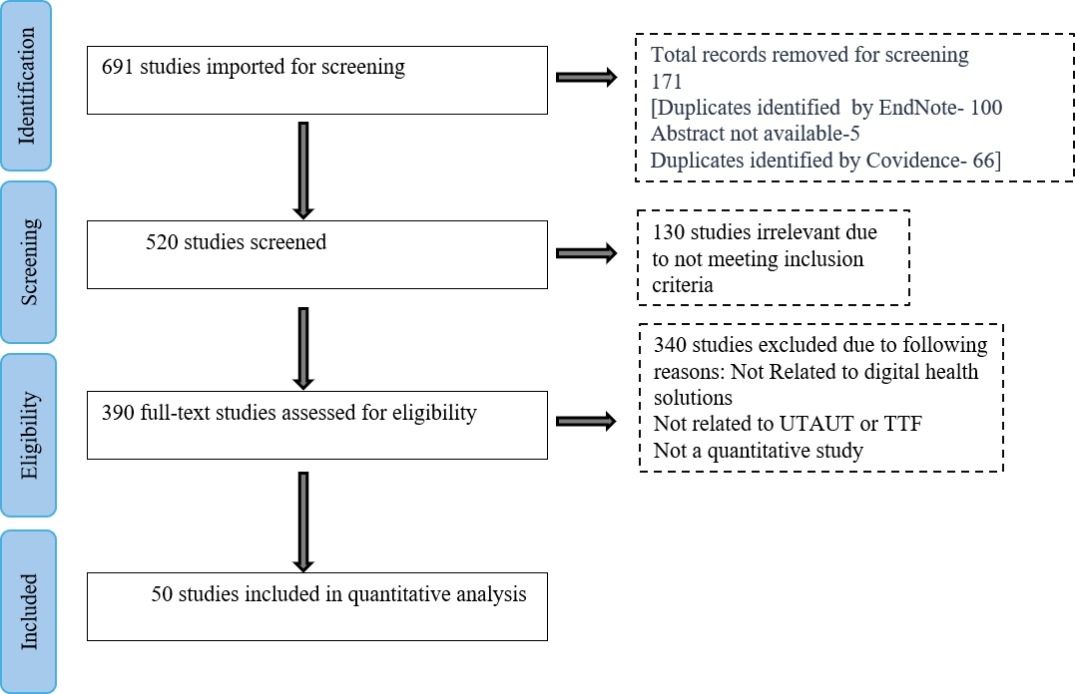
PRISMA (Preferred Reporting Items for Systematic Reviews and Meta-Analyses) flow diagram for the integrated Unified Theory of Acceptance and Use of Technology and task-technology fit systematic review and meta-analysis. TTF: task-technology fit; UTAUT: Unified Theory of Acceptance and Use of Technology.

The systematic review commenced with the identification of 691 potential studies from various databases, namely, IEEE (22/691, 3.2%), MEDLINE and Embase (134/691, 19.4%), ScienceDirect (149/691, 21.6%), Scopus (286/691, 41.4%), CINAHL (44/691, 6.4%), Google Scholar (18/691, 2.6%), and Web of Science (38/691, 5.5%). After removing duplicates (171/691, 24.8%), the titles and abstracts of 520 studies were screened. From these, 390 full-text articles were reviewed as eligible studies, resulting in 50 studies [11,12,15-62] (12.8% of the full-text articles assessed) being included in the final meta-analysis. The studies were excluded for not being related to digital health solutions, not relating to UTAUT or TTF, or not being quantitative studies (Figure 2).

**Figure 3.**
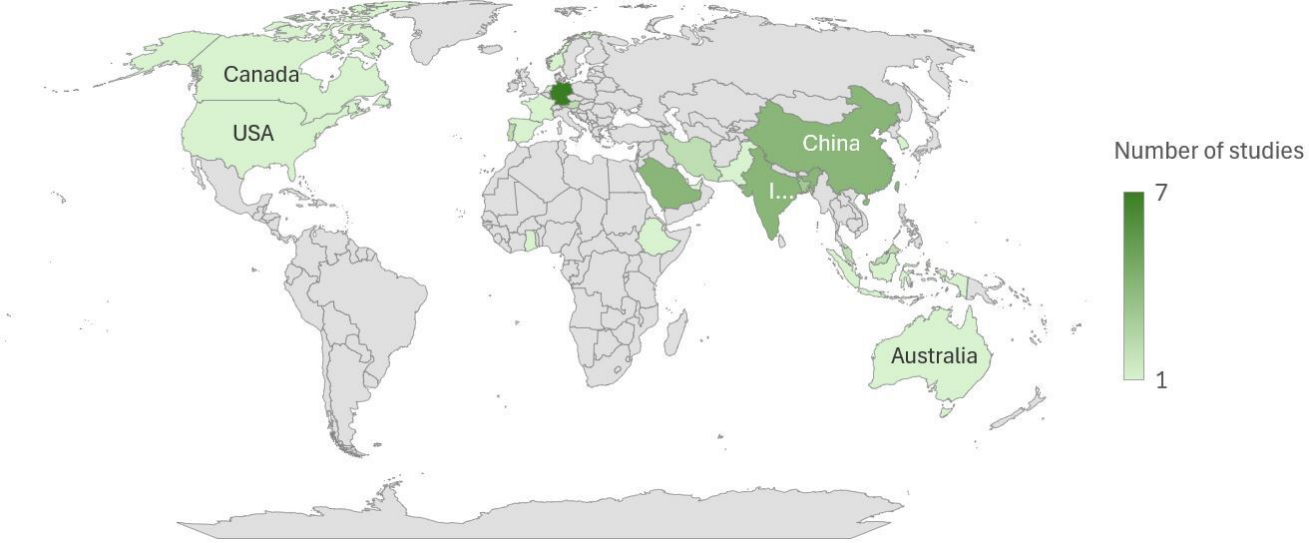
Distribution of articles by country.

The studies involved in this meta-analysis span various geographic regions (Figure 3) and use diverse methodologies to examine the factors influencing the adoption and acceptance of digital health technologies. The studies used a range of designs, including survey studies, cross-sectional analyses, and mixed methods approaches, and were conducted in various countries, highlighting the global interest and research efforts in understanding the adoption of digital health technologies.

Several demographic variables were reported across 50 different studies involving diverse populations. Most of the samples included individuals from diverse age groups, with all studies reporting age as a demographic variable. Gender was another frequently reported variable, present in 49 studies [11,12,15-59,61,73]. Education level was recorded in 20 studies [11,15,17,20,22,23,27,28,31,32,37-39,41,42,45,49,51,57,59], while marital and occupational status were less commonly reported, appearing in only 3 studies [32,36,37] and 7 studies [11,12,19,37,39,52,56], respectively. Some studies also recorded experience and work role, with 6 studies [25,26,33,43-45] each noting these variables. Other variables, such as income, familiarity with telemedicine, residence type, role, religion, nationality, chronic disease status, stage of disease, diabetic type, and disease duration, were also reported across the studies. This analysis emphasizes the need for comprehensive demographic profiling to better understand the diverse populations involved in studies in the health sector.

### Descriptive Analysis of Outcomes

Table 1 offers a detailed explanation of the 9 relationships between the variables within the health care context. The path coefficients exhibit significant variation across different studies. For instance, the path coefficients for PE to UI range from −0.023 to 0.753, for UI to UB range from −0.31 to 0.538, for SI to UI range from −0.088 to 0.718, and for EE to UI range from −0.280 to 0.884. Additionally, TTF-related paths range from −0.209 to 0.780 for TC-TTF, 0.199 to 0.780 for TechC-TTF, and −0.209 to 0.712 for TTF-UI. Additionally, the number of studies investigating each relationship varies notably. The PE-UI and EE-UI relationships were assessed in 35 studies [12,15-20,22-35,37-44,46-50,61], the FC-UI relationship in 31 studies [12,15-17,19,20,22-24,26-31,33-35,37-39,41-44,46-50,61], and the SI-UI relationship in 33 studies [12,15-20,22-34,37,39-44,46-50,61]. In comparison, only 17 studies [12,17,25-27,33,35,37,40-43,46,47,49,50,61] analyzed the UI-UB relationship, and 13 studies [16,25-27,33,35,38,41-43,46,47,61] examined the FC-UB link. The TC-TTF, TechC-TTF, and TTF-UI relationships were assessed in 14 [11,21,36,45,51-60], 15 [11,21,36,45,51-60,73], and 14 [11,21,36,45,51-53,55-60,73] studies, respectively.

Most empirical studies on health care technology adoption reported β values consistent with the UTAUT and TTF theories. Specifically, 86% of the PE-UI relationships, 74% of the EE-UI relationships, 71% of the UI-UB relationships, and 69% of the FC-UB relationships were positive and statistically significant. For TTF, 93% of TC-TTF, 100% of TechC-TTF, and 93% of TTF-UI relationships were positive and significant. However, there is variability in the significance and direction of some relationships. For instance, 64% of the observations for the SI-UI relationship were significant and positive, 24% were positive but not significant, and 12% were negative and nonsignificant.

**Table 1.** Summary of path coefficients, sample sizes, significance, and weight analysis.

Path	Total studies, N	Average (range)^a^	Sample size, N	Positive significance β, n (%)	Positive nonsignificance β, n (%)	Negative significance β, n (%)	Negative nonsignificance β, n (%)	Weight analysis significance β	Weight
PE^b^➔ UI^c^	35	0.285 (−0.023 to 0.753)	20,723	30 (86)	4 (11)	0 (0)	1 (3)	30	0.857
EE^d^➔ UI	35	0.198 (−0.280 to 0.884)	20,723	26 (74)	4 (11)	2 (6)	3 (9)	28	0.8
SI^e^➔ UI	33	0.160 (−0.088 to 0.718)	19,964	21 (64)	8 (24)	0 (0)	4 (12)	21	0.636
FC^f^➔ UI	31	0.118 (−0.153 to 0.548)	12,358	19 (61)	7 (23)	3 (10)	1 (3)	22	0.71
FC➔ UB^g^	13	0.192 (−0.110 to 0.561)	4943	9 (69)	3 (23)	1 (8)	0 (0)	10	0.769
UI➔ UB	17	0.218 (−0.310 to 0.538)	13,670	12 (71)	1 (6)	3 (18)	1 (6)	15	0.882
TechC^h^➔TTF^i^	15	0.438 (0.199 to 0.780)	4041	15 (100)	0 (0)	0 (0)	0 (0)	15	1
TC➔TTF	14	0.291 (−0.007 to 0.525)	3658	13 (93)	1 (7)	0 (0)	0 (0)	13	0.929
TTF➔ UI^j^	14	0.327 (−0.209 to 0.712)	3939	13 (93)	1 (7)	0 (0)	0 (0)	13	0.929

a Average β: arithmetic mean of β values.

b PE: performance expectancy.

c UI: usage intention.

d EE: effort expectancy.

e SI: social influence.

f FC: facilitating condition.

g UB: usage behavior.

h TechC: technology characteristic.

i TTF: task-technology fit.

j BI: behavioral intention.

### Weight Analysis Outcomes

Table 1 also presents a summary of the weight analysis for UTAUT and TTF-related relationships. The strength of each predictor was assessed based on 2 criteria, classifying them as either “experimental” or “well-used” [74]. Well-utilized predictors are extensively used and accepted in the field, backed by strong theory, substantial empirical evidence, and practical application. Experimental predictors are less common and newer to the field, often undergoing exploration and testing with limited empirical support and application in research and practice. In this study, all relationships were well-utilized, indicating that the behavioral determinants of PE, EE, SI, FC, and UI, and the task-technology alignment factors of TC, TechC, and TTF are commonly used constructs across studies related to health care technology.

A weight was assigned to each causal link by dividing the number of significant relationships (both positive and negative) by the total number of observations for that relationship [74]. The strongest predictors in the health care technology adoption literature were identified as PE on UI (weight=0.857), EE on UI (weight=0.800), and UI on UB (weight=0.882). For TTF, the strongest predictors were TechC on TTF (weight=1), TC on TTF (weight=0.929), and TTF on UI (weight=0.929), reflecting robust task-technology alignment.

### Meta-Analysis Outcomes

**Figure 4.**
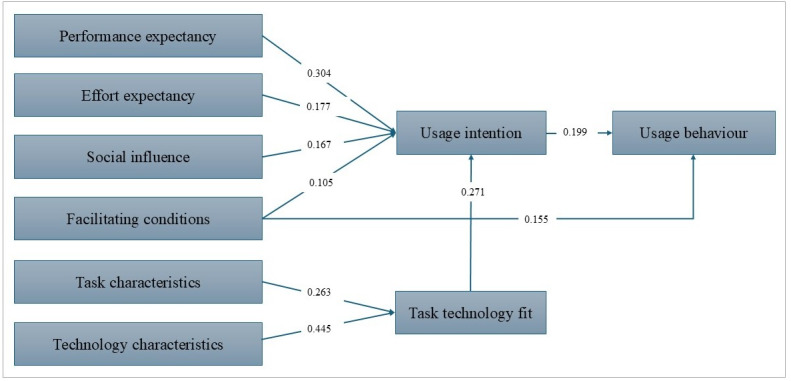
The meta-analytic outcomes.

Table 2 provides the effect sizes (weighted mean), significance levels (95% CIs). The meta-analysis confirms that all hypotheses (H1 through H6) are supported, demonstrating significant associations within the integrated UTAUT and TTF model for health care technology adoption. PE (H1: β=.304; *P*<.001) is a strong predictor of UI among health care professionals, with EE (H2: β=.177; *P*<.001) and SI (H3: β=.167; *P*<.001) also showing significant positive associations. Although the β-value for FC (H4: β=.105; *P*<.001) is relatively small, it suggests a likely significant association with UI. FC (H5: β=.155; *P*<.001) significantly influences UB, and UI (H6: β=.199; *P*<.001) emerges as the strongest predictor of actual UB among health care professionals. For TTF, TC (H7: β=.263; *P*<.001) and TechC (H8: β=.445; *P*<.001) positively influence TTF, which in turn predicts UI (H9: β=.271; *P*<.001), with TechC-TTF showing the strongest effect. Notably, the relationship between PE and UI (H1) is the strongest, followed by the relationship between UI and UB (H6). The weakest, though still significant, relationship is between FC and UI (H4). All constructs demonstrate good to excellent reliability and validity, as indicated by high average variance extracted, CR, and Cronbach α values, reinforcing the robustness of the findings and laying a strong foundation for future research and practical implementation in health care technology adoption (Figure 4).

With respect to estimating precision, some mean effect sizes were more precise than others. The 95% CIs for EE on UI (0.164‐0.191) and PE on UI (0.290‐0.317) were relatively narrow, indicating higher precision in estimating the mean effect sizes for the EE-UI and PE-UI relationships. Conversely, the 95% CI for FC on UB (0.127‐0.183) was wider, suggesting lower precision in the FC-UB estimate. Similarly, TC-TTF (0.231‐0.296) showed wider intervals, reflecting higher variability, while TechC-TTF (0.414‐0.476) indicated strong precision.

**Table 2.** Meta-analysis of path coefficients, total sample sizes, significance, and CI values.

Path	Number of occurrences	TSS^a^	Meta β (95% CI)^b^	Heterogeneity test		*P* value (β)
				Q	*I* ^2^	
PE^c^ ➔ UI^d^	35	20,723	0.304 (0.29-0.317)	223.2	85.2	<.001
EE^e^ ➔ UI	35	20,723	0.177 (0.164-0.191)	248	87.4	<.001
SI^f^ ➔ UI	33	19,964	0.167 (0.153-0.18)	304.6	89.1	<.001
FC^g^ ➔ UI	31	12,358	0.105 (0.088-0.123)	186.2	83.6	<.001
FC ➔ UB^h^	13	4943	0.155 (0.127-0.183)	70.9	81.9	<.001
UI ➔ UB	17	13,670	0.199 (0.183-0.216)	116.6	86.5	<.001
TechC^i^ ➔ TTF^j^	15	4041	0.445 (0.414-0.476)	155.6	91	<.001
TC^k^ ➔ TTF	14	3658	0.263 (0.231-0.296)	93.8	86.15	<.001
TTF ➔ BI^l^	14	3939	0.271 (0.237-0.299)	253.3	94.87	<.001

a TSS: total sample size.

b Meta *β*: weighted mean effect size.

c PE: performance expectancy.

d UI: usage intention.

e EE: effort expectancy.

f SI: social influence.

g FC: facilitating condition.

h UB: usage behavior.

i TechC: technology characteristic.

j TTF: task-technology fit.

k TC: task characteristic.

l BI: behavioral intention.

## Discussion

### Principal Findings

In the health care context of technology adoption, the results from the meta-analysis provide robust support for the integrated UTAUT and TTF model. PE was identified as a significant predictor of UI, indicating that health care professionals are more likely to adopt HITs if they perceive the technology will enhance their job performance. This finding aligns with previous studies by Hoque and Sorwar [27], Nadaf and Mousavi [38], and Diel et al [18], who found that perceived benefits significantly drive the intention to use health systems. PE’s stronger effect (β=.304; *P*<.001) compared to EE, SI, and FC likely reflects health care professionals’ prioritization of technologies that enhance job performance, such as EHRs improving patient care efficiency. This aligns with Zhou et al [63], who noted PE’s dominance in mHealth adoption. Our findings align with prior UTAUT-based research, such as Hoque and Sorwar [27], which identified PE as a consistent driver of UI in health care settings. However, the inconsistent effects of SI and FC, as noted in studies like García de Blanes Sebastián et al [31] and Araújo et al [34], may stem from contextual factors, such as cultural norms or organizational support levels. For instance, SI’s weaker effect in some studies could reflect settings where peer influence is less pronounced, such as individualistic cultures or noncollaborative health care environments. Similarly, FC’s variability may relate to differences in infrastructure availability, with resource-constrained settings showing weaker effects [14,17]. EE also showed a strong positive relationship with UI, suggesting that the perceived ease of using health care technologies influences willingness to adopt them. This is associated with research by Breil et al [13] and Farhady et al [26], which demonstrated that ease of use is crucial in technology acceptance among health care workers. SI was another important factor affecting UI. The findings suggest that health care professionals are more likely to adopt new technologies if they perceive these solutions as valued by important others (eg, peers and supervisors) and believe they should use the technology [39,40]. This aligns with the findings of Zhang et al [41] and Quaosar et al [42] who highlighted the role of SI in the adoption of HITs. FC significantly influenced both UI and UB. The availability of organizational and technical support systems appears to be important in encouraging health care professionals. Studies by Alharbi [33] and Walle et al [37] have similarly emphasized the importance of supportive infrastructure in technology adoption. UI was found to be the most critical predictor of actual UB. This relationship highlights the importance of understanding UI to predict the actual use of health technologies. The result is consistent with the technology acceptance literature on UI, shown to be the predominant predictor of UB in studies conducted with citizens using health information applications [43,44]. For TTF, technology characteristics (TechC) strongly predict TTF (β=.445; *P*<.001), with TTF influencing UI (β=.271; *P*<.001), as supported by Wang et al [11] and Hsieh and Lin [45]. This suggests that task-technology alignment enhances adoption, particularly in contexts like wearable devices and epidemic prevention systems, complementing UTAUT’s behavioral focus. The pooled meta-analytic findings reconcile discrepancies in individual studies by providing a weighted synthesis of effect sizes, revealing consistent positive effects for all UTAUT and TTF constructs. For example, while Arfi et al [24] found no PE-UI effect for IoT in eHealth, our meta-analysis confirms PE’s overall significance (β=.304; *P*<.001), suggesting context-specific anomalies. This enhances generalizability across diverse health care technologies (eg, EHRs and telemedicine), though high heterogeneity indicates caution in applying findings to resource-constrained or culturally distinct settings. The integration enhances understanding of system usefulness (PE and TTF), usability (EE and TC), and satisfaction (SI, FC, and UI), aligning with the proposed model.

### Theoretical Contributions

This meta-analysis makes several contributions to the existing body of literature, particularly on health care technology adoption. By confirming the applicability of the integrated UTAUT and TTF model, it strengthens the validity of the models in the health care context. The study extends previous research by including many empirical studies from the existing literature, reexamining the discrepancies, and providing a comprehensive synthesis of findings. It provides a strong understanding of the key factors influencing technology adoption, specifically in the health care setting. The integration reconciles UTAUT’s behavioral inconsistencies (eg, SI variability [14,17]) with TTF’s task-fit insights, offering a holistic framework validated by meta-analytic evidence. It provides a robust framework for understanding key adoption factors, enhancing insights into system usability, usefulness, and satisfaction.

### Managerial Implications

From a practical perspective, the findings recommend that health care organizations and policymakers focus on enhancing PE, EE, SI, and FC to promote health technology adoption. Training programs and administrative workflows should be tailored to meet diverse demographic needs to maximize adoption. For instance, a telemedicine adoption initiative in rural Australia successfully increased PE by demonstrating improved patient access to specialists, while tailored training programs enhanced EE among clinicians, leading to higher adoption rates. For example, younger professionals may benefit from advanced technical support, while educational initiatives can enhance adoption among less experienced staff. Additionally, organizations should prioritize TechC and TC alignment to optimize TTF, ensuring technologies meet task-specific needs, supporting efficient health care delivery, as shown in Taiwan-based studies.

### Limitations and Future Research Directions

This study has various limitations that should be addressed in future research. While the meta-analysis provides valuable insights, it is limited to studies published in English and available in selected databases. Future research could expand the scope to include various sources and languages. Additionally, this study focused on the core UTAUT and TTF constructs. Therefore, future research should explore other relevant factors, such as trust, perceived risk, security, and organizational culture. Moreover, this meta-analysis primarily considered quantitative studies. Including qualitative research could provide a richer understanding of the contextual factors influencing technology adoption. The high heterogeneity (*I*² 81.9%‐94.87%) suggests regional variations (eg, Taiwan vs Ethiopia), and future research should be planned to explore cultural influences. For example, collectivist cultures may emphasize SI more than individualistic ones, contributing to variability in SI-UI relationships, while differences in technological infrastructure, such as those between Taiwan and Ethiopia, may affect FC’s impact [14]. The lack of a formal risk of bias assessment is a limitation, and future studies should use the Joanna Briggs Institute or GRADE (Grading of Recommendations Assessment, Development and Evaluation) tools for transparency. The high heterogeneity also warrants subgroup analyses and meta-regression. Future work should also address publication bias using funnel plots and the Egger test. By addressing these limitations, future research can further enhance our understanding of technology adoption in health care settings and support the development of more effective strategies for promoting the use of innovative health technologies.

### Conclusions

Over the past 2 decades, research in the health care technology sector has grown significantly. However, the reported effect sizes in these studies often vary and sometimes contradict each other. To address these inconsistencies, this study conducted an SLR and meta-analysis, followed by a weight analysis based on findings related to UTAUT and TTF theories from 50 studies focused on health care settings. The descriptive analysis showed that PE, EE, and SI were the most used UTAUT variables in health care technology adoption studies, which integrated these factors into their theoretical models. TTF variables such as TechC and TTF were also prominent, enhancing the model’s scope. This meta-analysis’ results demonstrated that all path relationships in the integrated UTAUT and TTF model were statistically significant. Among the predictors, PE was identified as the strongest driver of UI compared to EE, SI, and FC. Furthermore, UI was identified as a stronger determinant of UB than facilitating conditions. TechC emerged as the strongest TTF driver, reflecting its role in task alignment. Theoretically, the meta-analysis advances the present knowledge on health care technology adoption. It provides a more cohesive understanding of the key factors influencing technology adoption in health care settings. For managers and policymakers, the findings highlight the importance of ensuring that health technologies are perceived as beneficial, easy to use, and socially recommended. Additionally, it is crucial to provide adequate support and training to meet the diverse needs of health care professionals. Ensuring TechC-TC fit can further boost adoption by aligning tools with tasks. These efforts will enhance technology adoption, thereby improving health care delivery and patient outcomes. This study offers valuable insights for both researchers and practitioners on how to promote the effective adoption of HIT.

## Supplementary material

10.2196/64524 Multimedia Appendix 1 Previous Unified Theory of Acceptance and Use of Technology and task technology fit studies.

10.2196/64524 Multimedia Appendix 2 Search strategy.

10.2196/64524 Checklist 1 PRISMA checklist.

## Abbreviations

CR: composite reliability
EE: effort expectancy
EHR: electronic health record
FC: facilitating condition
GRADE: Grading of Recommendations Assessment, Development and Evaluation
HIT: health information technology
IoT: Internet of Things
mHealth: mobile health
PE: performance expectancy
PRISMA: Preferred Reporting Items for Systematic Reviews and Meta-Analyses
SI: social influence
SLR: systematic literature review
TC: task characteristic
TechC: technology characteristic
TTF: task-technology fit
UB: usage behavior
UI: usage intention
UTAUT: Unified Theory of Acceptance and Use of Technology

## References

[R1] Venkatesh V, Morris MG, Davis GB, Davis FD (2003). User acceptance of information technology: toward a unified view. MIS Q.

[2] Goodhue DL, Thompson RL (1995). Task-technology fit and individual performance. MIS Q.

[3] Tavares J, Goulão A, Oliveira T (2018). Electronic health record portals adoption: empirical model based on UTAUT2. Inform Health Soc Care.

[4] Al Moteri M, Alojail M (2023). Factors influencing the supply chain management in e-Health using UTAUT model. ERA.

[5] Zheng R, Jiang X, Shen L (2025). Investigating clinicians’ intentions and influencing factors for using an intelligence-enabled diagnostic clinical decision support system in health care systems: cross-sectional survey. J Med Internet Res.

[6] Cimperman M, Makovec Brenčič M, Trkman P (2016). Analyzing older users’ home telehealth services acceptance behavior-applying an extended UTAUT model. Int J Med Inform.

[7] Barchielli C, Marullo C, Bonciani M, Vainieri M (2021). Nurses and the acceptance of innovations in technology-intensive contexts: the need for tailored management strategies. BMC Health Serv Res.

[8] Neeragatti S, Dehury RK, Sripathi N (2023). Determinants of Digital Health Information Search (DHIS) behaviour: extending UTAUT with healthcare behaviour constructs. APJHM.

[9] Alshahrani A, Williams H, MacLure K (2022). Investigating health managers’ perspectives of factors influencing their acceptance of eHealth services in the Kingdom of Saudi Arabia: a quantitative study. Saudi J Health Syst Res.

[10] Garavand A, Samadbeik M, Nadri H, Rahimi B, Asadi H (2019). Effective factors in adoption of mobile health applications between medical sciences students using the UTAUT model. Methods Inf Med.

[11] Wang H, Tao D, Yu N, Qu X (2020). Understanding consumer acceptance of healthcare wearable devices: An integrated model of UTAUT and TTF. Int J Med Inform.

[12] Candra S, Williar AY, Princes E, Loang OK, Delphin G, Basmantra IN Using an extended UTAUT theory to examine the consumer behavior of m-health apps: preliminary results.

[13] Breil B, Kremer L, Hennemann S, Apolinário-Hagen J (2019). Acceptance of mHealth apps for self-management among people with hypertension. Stud Health Technol Inform.

[14] Williams MD, Rana NP, Dwivedi YK (2015). The unified theory of acceptance and use of technology (UTAUT): a literature review. J Enterp Inf Manag.

[15] Duarte P, Pinho JC (2019). A mixed methods UTAUT2-based approach to assess mobile health adoption. J Bus Res.

[16] Thabet Z, Albashtawi S, Ansari H, Al-Emran M, Al-Sharafi MA, AlQudah AA (2023). Exploring the factors affecting telemedicine adoption by integrating UTAUT2 and IS success model: a hybrid SEM–ANN approach. IEEE Trans Eng Manage.

[17] Dash A, Sahoo AK (2022). Exploring patient’s intention towards e-health consultation using an extended UTAUT model. JET.

[18] Diel S, Doctor E, Reith R, Buck C, Eymann T (2023). Examining supporting and constraining factors of physicians’ acceptance of telemedical online consultations: a survey study. BMC Health Serv Res.

[19] Qvist A, Mullan L, Nguyen L (2024). Investigating allied health professionals’ attitudes, perceptions and acceptance of an electronic medical record using the Unified Theory of Acceptance and Use of Technology. Aust HEALTH Rev.

[20] Dadhich M, Hiran KK, Rao SS, Sharma R (2022). Factors influencing patient adoption of the IoT for E-Health Management Systems (e-HMS) using the UTAUT model. IJACI.

[21] Alkhalifah A, Bukar UA (2022). Examining the prediction of COVID-19 contact-tracing app adoption using an integrated model and hybrid approach analysis. Front Public Health.

[22] Tian XF, Wu RZ (2022). Determinants of the mobile health continuance intention of elders with chronic diseases: an integrated framework of ECM-ISC and UTAUT. Int J Environ Res Public Health.

[23] Schomakers EM, Lidynia C, Vervier LS, Calero Valdez A, Ziefle M (2022). Applying an extended UTAUT2 model to explain user acceptance of lifestyle and therapy mobile health apps: survey study. JMIR Mhealth Uhealth.

[24] Arfi WB, Nasr IB, Kondrateva G, Hikkerova L (2021). The role of trust in intention to use the IoT in eHealth: application of the modified UTAUT in a consumer context. Technol Forecast Soc Change.

[25] Venugopal S (2018). An analysis of the impact of UTAUT predictors on the intention and usage of electronic health records and telemedicine from the perspective of clinical staffs. IJOMAM.

[26] Farhady S, Sepehri MM, Pourfathollah AA (2020). Evaluation of effective factors in the acceptance of mobile health technology using the Unified Theory of Acceptance and Use of Technology (UTAUT), case study: blood transfusion complications in thalassemia patients. Med J Islam Repub Iran.

[27] Hoque R, Sorwar G (2017). Understanding factors influencing the adoption of mHealth by the elderly: an extension of the UTAUT model. Int J Med Inform.

[28] Lathifah A, Putro US, Rinawan FR, Novani S, Hasyimi V, Tiara AR (2023). Understanding participation in value co-creation and acceptance of iPosyandu by extending UTAUT among community health workers. CommIT.

[29] Schmitz A, Díaz-Martín AM, Yagüe Guillén MJ (2022). Modifying UTAUT2 for a cross-country comparison of telemedicine adoption. Comput Human Behav.

[30] Napitupulu D, Yacub R, Putra A (2021). Factor influencing of telehealth acceptance during COVID-19 outbreak: extending UTAUT model. IJIES.

[31] García de Blanes Sebastián M, Sarmiento Guede JR, Antonovica A (2022). Application and extension of the UTAUT2 model for determining behavioral intention factors in use of the artificial intelligence virtual assistants. Front Psychol.

[32] Nurtsch A, Teufel M, Jahre LM (2024). Drivers and barriers of patients’ acceptance of video consultation in cancer care. Digit Health.

[33] Alharbi F (2021). The use of digital healthcare platforms during the COVID-19 pandemic: the consumer perspective. Acta Inform Med.

[34] Araújo I, Grilo A, Silva C (2023). Portuguese validation of the Unified Theory of Acceptance and Use of Technology Scale (UTAUT) to a COVID-19 mobile application: a pilot study. Healthcare (Basel).

[35] Owusu Kwateng K, Darko-Larbi O, Amanor K (2023). A modified UTAUT2 for the study of telemedicine adoption. Int J Healthc Manag.

[36] Kang HJ, Han J, Kwon GH (2022). The acceptance behavior of smart home health care services in South Korea: an integrated model of UTAUT and TTF. Int J Environ Res Public Health.

[37] Walle AD, Jemere AT, Tilahun B (2023). Intention to use wearable health devices and its predictors among diabetes mellitus patients in Amhara region referral hospitals, Ethiopia: using modified UTAUT-2 model. Inform Med Unlocked.

[38] Nadaf M, Mousavi SJ (2022). Using UTAUT2 model for explaining telemedicine adoption, evidence from Iran. IOH.

[39] Alomari A, Soh B (2023). Determinants of medical internet of things adoption in healthcare and the role of demographic factors incorporating modified UTAUT. IJACSA.

[40] Gansser OA, Reich CS (2021). A new acceptance model for artificial intelligence with extensions to UTAUT2: an empirical study in three segments of application. Technol Soc.

[41] Zhang Y, Liu C, Luo S (2019). Factors influencing patients’ intentions to use diabetes management apps based on an extended Unified Theory of Acceptance and Use of Technology model: web-based survey. J Med Internet Res.

[42] Quaosar G, Hoque MR, Bao Y (2018). Investigating factors affecting elderly’s intention to use m-Health services: an empirical study. Telemed J E Health.

[43] Bile Hassan I, Murad MAA, El-Shekeil I, Liu J (2022). Extending the UTAUT2 model with a privacy calculus model to enhance the adoption of a health information application in Malaysia. Informatics (MDPI).

[44] Seethamraju R, Diatha KS, Garg S (2018). Intention to use a mobile-based information technology solution for tuberculosis treatment monitoring – applying a UTAUT model. Inf Syst Front.

[45] Hsieh PJ, Lin WS (2020). Understanding the performance impact of the epidemic prevention cloud: an integrative model of the task-technology fit and status quo bias. Behav Inf Technol.

[46] Schretzlmaier P, Hecker A, Ammenwerth E (2023). Predicting mHealth acceptance using the UTAUT2 technology acceptance model: a mixed-methods approach. Stud Health Technol Inform.

[47] van Bussel MJP, Odekerken-Schröder GJ, Ou C, Swart RR, Jacobs MJG (2022). Analyzing the determinants to accept a virtual assistant and use cases among cancer patients: a mixed methods study. BMC Health Serv Res.

[48] Baum U, Kühn F, Lichters M (2022). Neurological outpatients prefer EEG home-monitoring over inpatient monitoring-an analysis based on the UTAUT model. Int J Environ Res Public Health.

[49] Barua Z, Barua A (2021). Acceptance and usage of mHealth technologies amid COVID-19 pandemic in a developing country: the UTAUT combined with situational constraint and health consciousness. JET.

[50] Zhu Y, Zhao Z, Guo J (2023). Understanding use intention of mHealth applications based on the Unified Theory of Acceptance and Use of Technology 2 (UTAUT-2) model in China. Int J Environ Res Public Health.

[51] Wang SL, Lin HI (2019). Integrating TTF and IDT to evaluate user intention of big data analytics in mobile cloud healthcare system. Behav Inf Technol.

[52] Alhendawi KM (2024). Task-technology fit model: modelling and assessing the nurses’ satisfaction with health information system using AI prediction models. Int J Healthc Manag.

[53] Abdekhoda M, Dehnad A, Zarei J (2022). Factors influencing adoption of e-learning in healthcare: integration of UTAUT and TTF model. BMC Med Inform Decis Mak.

[54] O’Connor Y, Andreev P, O’Reilly P (2020). MHealth and perceived quality of care delivery: a conceptual model and validation. BMC Med Inform Decis Mak.

[55] Wijaya L, Ng KC, Sihombing PR Assessing determinants of the telemedicine applications continuance usage intention with TTF theory.

[56] El-Masri M, Al-Yafi K, Kamal MM (2023). A task-technology-identity fit model of smartwatch utilisation and user satisfaction: a hybrid SEM-neural network approach. Inf Syst Front.

[57] Lin TC (2014). Mobile nursing information system utilization: the task-technology fit perspective. Comput Inform Nurs.

[58] Yamin MAY, Alyoubi BA (2020). Adoption of telemedicine applications among Saudi citizens during COVID-19 pandemic: an alternative health delivery system. J Infect Public Health.

[59] Shahbaz M, Gao C, Zhai L, Shahzad F, Khan I (2021). Environmental air pollution management system: predicting user adoption behavior of big data analytics. Technol Soc.

[60] Hsiao JL, Chen RF (2012). An investigation on task-technology fit of mobile nursing information systems for nursing performance. Comput Inform Nurs.

[61] Schretzlmaier P, Hecker A, Ammenwerth E (2022). Extension of the Unified Theory of Acceptance and Use of Technology 2 model for predicting mHealth acceptance using diabetes as an example: a cross-sectional validation study. BMJ Health Care Inform.

[62] Wu P, Zhang RT, Zhu XM, Liu ML (2022). Factors influencing continued usage behavior on mobile health applications. Healthcare (Basel).

[63] Zhou J, Liu Z, Li J, Jiao H Technology complementarity and collaborative innovation: the moderating effects of IT adoption.

[64] Hennington A, Janz BD (2007). Information systems and healthcare XVI: physician adoption of electronic medical records: applying the UTAUT model in a healthcare context. CAIS.

[65] Kim YJ, Choi JH, Fotso GMN (2024). Medical professionals’ adoption of AI-based medical devices: UTAUT model with trust mediation. Journal of Open Innovation: Technology, Market, and Complexity.

[66] Jewer J (2018). Patients’ intention to use online postings of ED wait times: a modified UTAUT model. Int J Med Inform.

[67] Cao J, Kurata K, Lim Y, Sengoku S, Kodama K (2022). Social acceptance of mobile health among young adults in Japan: an extension of the UTAUT model. Int J Environ Res Public Health.

[68] Lee WI, Fu HP, Mendoza N, Liu TY (2021). Determinants impacting user behavior towards emergency use intentions of m-Health services in Taiwan. Healthcare (Basel).

[69] Alam MZ, Hu W, Kaium MA, Hoque MR, Alam MMD (2020). Understanding the determinants of mHealth apps adoption in Bangladesh: a SEM-Neural network approach. Technol Soc.

[70] Dwivedi YK, Rana NP, Jeyaraj A, Clement M, Williams MD (2019). Re-examining the Unified Theory of Acceptance and Use of Technology (UTAUT): towards a revised theoretical model. Inf Syst Front.

[71] Baptista G, Oliveira T (2016). A weight and a meta-analysis on mobile banking acceptance research. Comput Human Behav.

[72] El Kurdi B, Babar S, El Iskandarani M (2019). Factors that affect prevalence of small intestinal bacterial overgrowth in chronic pancreatitis: a systematic review, meta-analysis, and meta-regression. Clin Transl Gastroenterol.

[73] Al-Rahmi AM, Shamsuddin A, Wahab E (2022). Integrating the role of UTAUT and TTF model to evaluate social media use for teaching and learning in higher education. Front Public Health.

[74] Rana NP, Dwivedi YK, Williams MD (2015). A meta-analysis of existing research on citizen adoption of e-government. Inf Syst Front.

