# Supplementary material for: Determinants of Health Care Technology Adoption Using an Integrated Unified Theory of Acceptance and Use of Technology and Task Technology Fit Model: Systematic Review and Meta-Analysis
Source: J Med Internet Res. 2025 Dec 30;27:e64524. doi: 10.2196/64524 (PMC12753102; doi:10.2196/64524)
Supplement: Multimedia Appendix 1 [file jmir-v27-e64524-s001.docx]

**Table S1.** Study summary.

| **No** | **Author** | **Year** | **Title** | **Journal** | **Key Findings** |
| --- | --- | --- | --- | --- | --- |
| 1 | Hoque and Sorwar, 2017 [27] | 2017 | Understanding factors influencing the adoption of mHealth by the elderly: An extension of the UTAUT model | International Journal of Medical Informatics | Performance Expectancy, Effort Expectancy, Social Influence significantly impact Behavioral Intention to use mHealth |
| 2 | Diel et al., 2023 [18] | 2023 | Examining supporting and constraining factors of physicians’ acceptance of telemedical online consultations: a survey study | BMC Health Services Research | Performance expectancy, effort expectancy, and social influence significantly affect the intention to use telemedicine. IT anxiety negatively impacts performance and effort expectancy. Data security is crucial for acceptance. |
| 3 | Zhang et al., 2023 [62] | 2023 | Using Utaut2 Model For Explaining Telemedicine Adoption, Evidence From Iran | Iran Occupational Health | Performance expectancy, effort expectancy, facilitating conditions, hedonic motivation, perceived product advantage, and perceived security positively impact the intention to use telemedicine. The moderating role of innovativeness and gender was confirmed. |
| 4 | Zhang et al., 2023 [41] | 2019 | Factors Influencing Patients’ Intentions to Use Diabetes Management Apps Based on an Extended Unified Theory of Acceptance and Use of Technology Model: Web-Based Survey | Journal of Medical Internet Research | Performance expectancy and social influence are the strongest determinants of behavioral intention. Facilitating conditions and perceived privacy risk also impact intention. |
| 5 | Nurtsch et al., 2024 [32] | 2024 | Drivers and barriers of patients’ acceptance of video consultation in cancer care | Digital Health | Acceptance of video consultations is high among cancer patients. Significant predictors include younger age, female gender, advanced stage of disease, high digital confidence, low internet anxiety, moderate digital overload, high eHealth literacy, high personal trust, frequent internet use, and UTAUT predictors: performance expectancy, effort expectancy, and social influence. |
| 6 | Quaosar et al., 2018 [42] | 2018 | Investigating Factors Affecting Elderly’s Intention to Use m-Health Services: An Empirical Study | Telemedicine and e-Health | Performance expectancy, effort expectancy, social influence, and perceived credibility significantly influence the intention to use m-health services among the elderly. Facilitating conditions did not have a significant effect. |
| 7 | Schretzlmaier et al., 2023 [46] | 2023 | Predicting mHealth Acceptance Using the UTAUT2 Technology Acceptance Model: A Mixed-Methods Approach | dHealth | Performance expectancy, habit, perceived disease threat, and trust significantly predict behavioral intention to use mHealth apps. The extended UTAUT2 model explained 35% of the variance in behavioral intention. |
| 8 | Schomakers et al., 2022 [23] | 2022 | Applying an Extended UTAUT2 Model to Explain User Acceptance of Lifestyle and Therapy Mobile Health Apps: Survey Study | JMIR Mhealth Uhealth | The UTAUT2 model weakly predicted the intention to use mHealth apps. Hedonic motivation significantly predicted behavioral intentions for both app types. Habit influenced lifestyle apps, while social influence and trust influenced therapy apps. Health app familiarity showed the strongest correlation with the intention to use |
| 9 | Farhady et al., 2020 [26] | 2020 | Evaluation of effective factors in the acceptance of mobile health technology using the unified theory of acceptance and use of technology (UTAUT), case study: Blood transfusion complications in thalassemia patients | Medical Journal of the Islamic Republic of Iran | Performance expectancy and effort expectancy significantly affect behavioral intention. Usability and knowledge of IT also significantly affect performance expectancy. Facilitating conditions significantly affect use behavior. |
| 10 | van Bussel et al., 2022 [47] | 2022 | Analyzing the determinants to accept a virtual assistant and use cases among cancer patients: a mixed methods study | BMC Health Services Research | Performance expectancy (ß = 0.399), effort expectancy (ß = 0.258), social influence (ß = 0.114), and trust (ß = 0.210) significantly influence behavioral intention to use a virtual assistant. Self-efficacy (ß = 0.792) affects effort expectancy. |
| 11 | Alharbi, 2021 [33] | 2021 | The Use of Digital Healthcare Platforms During the COVID-19 Pandemic: the Consumer Perspective | Acta Informatica Medica | Facilitating conditions (t=0.233, p=0.023) and trust (t=0.324, p=0.005) had a significant impact on consumers’ behavioral intention of using digital healthcare platforms during the COVID-19 pandemic. Performance expectancy and effort expectancy were not significant. |
| 12 | Lathifah et al., 2023 [28] | 2023 | Understanding Participation in Value Co-Creation and Acceptance of iPosyandu by Extending UTAUT among Community Health Workers | CommIT Journal | Effort expectancy (β=0.623, p=0.001), perceived policy support (β=0.192, p=0.007), and intention to participate in value co-creation (β=0.206, p=0.005) significantly affect the intention to use iPosyandu. Performance expectancy, social influence, and facilitating conditions are not significant. |
| 13 | Duarte and Pinho, 2019 [15] | 2019 | A mixed methods UTAUT2-based approach to assess mobile health adoption | Journal of Business Research | Performance expectancy, hedonic motivation, and habit significantly predict mHealth adoption in PLS-SEM analysis. fsQCA reveals six configurations including these factors. Education level shows a significant positive effect on mHealth adoption. |
| 14 | Baum et al., 2022 [48] | 2022 | Neurological Outpatients Prefer EEG Home-Monitoring over Inpatient Monitoring—An Analysis Based on the UTAUT Model | International Journal of Environmental Research and Public Health | Multiple configurations lead to positive impacts including reliable and user-friendly technology, support from colleagues, and training. Negative impacts arise from lack of reliability, user-friendliness, training, and planning involvement. Leadership responsibilities also influence outcomes. |
| 15 | Walle et al., 2023 [37] | 2023 | Intention to use wearable health devices and its predictors among diabetes mellitus patients in Amhara region referral hospitals, Ethiopia | Informatics in Medicine Unlocked | Intention to use wearable health devices was 47.1%. Significant predictors include effort expectancy (β = 0.543, P < 0.01), performance expectancy (β = 0.306, P < 0.01), facilitating condition (β = 0.131, P < 0.05), and habit (β = 0.093, P < 0.05). Effort expectancy was positively moderated by gender (β = 0.780, p < 0.001) and performance expectancy by age (β = 0.439, p < 0.001). Social influence, hedonic motivation, and price value did not significantly influence the intention to use. |
| 16 | Alomari and Soh et al., 2023 [39] | 2023 | Determinants of Medical Internet of Things Adoption in Healthcare and the Role of Demographic Factors Incorporating Modified UTAUT | International Journal of Advanced Computer Science and Applications | The study identified that Computer and English language Self-Efficacy (CESE), Performance Expectancy (PE), and Social Influence (SI) are significant determinants for the adoption of mIoT. CESE showed the strongest influence. |
| 17 | Gansser and Reich et al., 2021 [40] | 2021 | A new acceptance model for artificial intelligence with extensions to UTAUT2: An empirical study in three segments of application | Technology in Society | Health, convenience comfort, and sustainability positively influence performance expectancy. - Performance expectancy, safety security (negative), personal innovativeness, effort expectancy, social influence, price value, hedonic motivation, and habit influence behavioral intention. - Habit and behavioral intention influence use behavior. - Safety security does not significantly influence behavioral intention in the health segment.C20 |
| 18 | Qvist et al., 2024 [19] | 2024 | Investigating allied health professionals' attitudes, perceptions, and acceptance of an electronic medical record using the Unified Theory of Acceptance and Use of Technology | Australian Health Review | AHPs had positive attitudes towards EMR use both pre- and post-implementation. Post-implementation, they felt more positive about system ease of use and had decreased anxiety. They reported adequate resources and knowledge to use EMR, with real-time data accessibility as a main advantage. Disadvantages included an unfriendly user interface, system outages, and decreased efficiency. Overall, increased EMR system familiarity improved positivity towards its use. |
| 19 | Dadhich et al., 2023 [20] | 2023 | Quantifying the Dynamic Factors Influencing New-Age Users’ Adoption of 5G Using TAM and UTAUT Models in Emerging Country: A Multistage PLS-SEM Approach | Education Research International | Users’ perceptions of adopting 5G are overwhelmingly positive, with perceived trust being a significant mediator between behavioral intention and various factors such as performance expectancy, effort expectancy, social factors, facilitating factors, hedonic motivation, perceived benefits, price value, and habit. |
| 20 | Schretzlmaier et al., 2022 [61] | 2022 | Extension of the Unified Theory of Acceptance and Use of Technology 2 model for predicting mHealth acceptance using diabetes as an example: a cross-sectional validation study | BMJ Health Care Inform | The extended UTAUT2 model, including perceived disease threat and trust, explains 35.0% of the variance in behavioral intention to use mobile diabetes applications. |
| 21 | Barua et al., 2021 [49] | 2021 | Acceptance and usage of mHealth technologies amid COVID-19 pandemic in a developing country: the UTAUT combined with situational constraint and health consciousness | Journal of Enabling Technologies | Situational constraint and health consciousness have strong direct positive effects on both behavioral intention and use behavior. Effort expectancy is insignificant in both direct and interaction effects. Social influence is significant in direct effects but insignificant in interaction effects. |
| 22 | Dash and Sahoo et al., 2022 [17] | 2022 | Exploring patient’s intention towards e-health consultation using an extended UTAUT model | Journal of Enabling Technologies | Positive and significant impact of PE, EE, trust, and SI on BI; FC and PR not significantly connected to BI |
| 23 | Venugopal et al., 2018 [25] | 2018 | An Analysis of the Impact of UTAUT Predictors on the Intention and Usage of Electronic Health Records and Telemedicine | International Journal of Mechatronics and Applied Mechanics | Performance expectancy, effort expectancy, and social influence significantly impact behavioral intention. Facilitating conditions significantly impact usage behavior. |
| 24 | Napitupulu et al., 2021 [30] | 2021 | Factor Influencing of Telehealth Acceptance During COVID-19 Outbreak: Extending UTAUT Model | International Journal of Intelligent Engineering and Systems | Performance expectancy, effort expectancy, and facilitating conditions significantly affect behavioral intention to use Telehealth. Social Influence is not significantly associated with behavioral intention. |
| 25 | Thabet et al., 2023 [16] | 2023 | Exploring the Factors Affecting Telemedicine Adoption by Integrating UTAUT2 and IS Success Model: A Hybrid SEM–ANN Approach | IEEE Transactions on Engineering Management | Performance expectancy, hedonic motivation, perceived security, and user satisfaction significantly drive telemedicine adoption. Effort expectancy, social influence, and facilitating conditions do not significantly impact telemedicine adoption. User satisfaction is the most important driver for telemedicine adoption. |
| 26 | Zhu et al., 2023 [50] | 2023 | Understanding Use Intention of mHealth Applications Based on the Unified Theory of Acceptance and Use of Technology 2 (UTAUT-2) Model in China | International Journal of Environmental Research and Public Health | Performance expectancy, effort expectancy, social influence, facilitating condition, and perceived trust positively affect use intention. Perceived risk negatively affects use intention. Price value has no significant effect. |
| 27 | Arfi et al., 2021 [24] | 2021 | The role of trust in intention to use the IoT in eHealth: Application of the modified UTAUT in a consumer context | Technological Forecasting & Social Change | Performance expectancy has no impact on intention to use the IoT for eHealth. Social Influence and Facilitating Conditions are significant predictors of Behavioral Intention. Perceived Trust negatively influences Perceived Risk, which in turn negatively influences Behavioral Intention. |
| 28 | Schmitz et al., 2022 [29] | 2022 | Modifying UTAUT2 for a cross-country comparison of telemedicine adoption | Computers in Human Behavior | Performance expectancy, hedonic motivation, perceived security, and perceived product advantage positively and significantly impact the behavioral intention to use virtual doctor appointments. Effort expectancy, social influences, facilitating conditions, and habit were not statistically significant. |
| 29 | Kwateng et al., 2023 [35] | 2023 | A modified UTAUT2 for the study of telemedicine adoption | International Journal of Healthcare Management | Performance Expectancy, Habit, and Hedonic Motivation influence behavioral intention to adopt telemedicine. Facilitating Conditions impact Use Behavior. |
| 30 | Araújo et al., 2023 [34] | 2023 | Portuguese Validation of the Unified Theory of Acceptance and Use of Technology Scale (UTAUT) to a COVID-19 Mobile Application: A Pilot Study | Healthcare | The Portuguese version of the UTAUT model showed good reliability (Cronbach’s α = 0.82) and acceptable overall adjustment to the sample. The initial model indicated an acceptable model fit (χ2/df = 3.732, RMSEA = 0.05, CFI = 0.955, TLI = 0.944, SRMR = 0.06), which improved after modifications. Performance expectancy and social influence were significant predictors of behavioral intention. The constructs effort expectancy, facilitating conditions, and innovativeness did not demonstrate convergent validity in this sample. The study highlighted the importance of performance and social influence in technology adoption. |
| 31 | Seethamraju et al., 2018 [44] | 2018 | Intention to Use a Mobile-Based Information Technology Solution for Tuberculosis Treatment Monitoring – Applying a UTAUT Model | Information Systems Frontiers | PE, EE, SI, and FC significantly and positively influence healthcare professionals’ behavioral intention to use the proposed solution, explaining 56% of the variance in BI. |
| 32 | Bile Hassan et al., 2022 [43] | 2022 | Extending the UTAUT2 Model with a Privacy Calculus Model to Enhance the Adoption of a Health Information Application in Malaysia | Informatics | Behavioral intentions significantly predict use behavior.   - Facilitating conditions significantly affect use behavior but not behavioral intentions.   Habit significantly influences both behavioral intentions and use behavior.  - Effort expectancy, performance expectancy, social influence, hedonic motivation, and price value significantly influence behavioral intentions.   - Perceived risk and privacy concern significantly affect use behavior. <  - Trust in SNIC does not significantly affect use behavior but significantly influences performance expectancy. |
| 33 | Candra and Williar, 2022 [12] | 2022 | Using an Extended UTAUT Theory to Examine The Consumer Behavior of M-Health Apps: Preliminary Results | IEEE | Performance expectancy, effort expectancy, social influence, and facilitating conditions significantly impact behavioral intention and actual usage behavior of m-health apps. Effort expectancy has a significant impact on behavioral intention. |
| 34 | García de Blanes Sebastián et al., 2022 [31] | 2022 | Application and extension of the UTAUT2 model for determining behavioral intention factors in use of the artificial intelligence virtual assistants | Frontiers in Psychology | Habit, trust, and personal innovation significantly impact the adoption of virtual assistants; Performance expectancy, effort expectancy, facilitating conditions, social influence, hedonic motivation, price/value, and perceived privacy risk were not significant factors |
| 35 | Tian and Wu, 2022 [22] | 2022 | Determinants of the Mobile Health Continuance Intention of Elders with Chronic Diseases: An Integrated Framework of ECM-ISC and UTAUT | International Journal of Environmental Research and Public Health | Confirmation significantly influences satisfaction, performance expectancy, and effort expectancy. Performance expectancy has the most significant direct influence on continuance intention. Social influence and facilitating conditions also significantly influence continuance intention. |
| 36 | Wang et al., 2020 [11] | 2020 | Understanding Consumer Acceptance of Healthcare Wearable Devices: An Integrated Model of UTAUT and TTF | International Journal of Medical Informatics | The study shows that performance expectancy, effort expectancy, social influence, facilitating conditions, and task-technology fit positively influence the acceptance of healthcare wearable devices (HWDs). These factors explained 68% of the variance in behavioral intention to use HWDs. |
| 37 | Wang and Lin, 2019 [51] | 2019 | Integrating TTF and IDT to evaluate user intention of big data analytics in mobile cloud healthcare system | Behaviour & Information Technology | Task characteristics, technology characteristics, and relative advantage positively influence task-technology fit, which in turn influences intention to use the mobile healthcare system. Observability had no significant effect. |
| 38 | Alhendawi, 2022 [52] | 2022 | Task-technology fit model: Modelling and assessing the nurses’ satisfaction with health information system using AI prediction models | International Journal of Healthcare Management | Task and technology characteristics, along with nurses' attitudes and task-technology fit, influence nurses' satisfaction with HIS. ANN model outperforms regression in predictive accuracy. |
| 39 | Abdekhoda et al., 2022 [53] | 2022 | Factors influencing adoption of e-learning in healthcare: integration of UTAUT and TTF model | BMC Medical Informatics and Decision Making | Technology and task characteristics, task-technology fit, social influences, effort expectancy, performance expectancy, and facilitating conditions positively influence e-learning adoption. |
| 40 | O’Connor et al., 2020 [54] | 2020 | MHealth and perceived quality of care delivery: a conceptual model and validation | BMC Medical Informatics and Decision Making | Task-technology fit (TTF) and mHealth utilization positively impact physicians' perceived quality of care (PQoC). Self-efficacy is crucial in mHealth utilization. |
| 41 | Alkhalifah and Bukar, 2022 [21] | 2022 | Examining the Prediction of COVID-19 Contact-Tracing App Adoption Using an Integrated Model and Hybrid Approach Analysis | Frontiers in Public Health | Perceived ease of use, usefulness, task features, and mobility positively impact intention to adopt the app; privacy risk was not significant |
| 42 | Wijaya et al., 2023 [55] | 2023 | Assessing Determinants of the Telemedicine Applications Continuance Usage Intention with TTF Theory | 8th International Conference on Business and Industrial Research | Technology characteristics and self-efficacy significantly impact telemedicine continuance usage, while task characteristics and awareness have minimal effects. |
| 43 | El-Masri et al., 2023 [56] | 2023 | A Task-Technology-Identity Fit Model of Smartwatch Utilisation and User Satisfaction | Information Systems Frontiers | Task-technology fit and technology-identity fit both significantly affect user satisfaction with smartwatches. Identity fit has a stronger effect on satisfaction, while actual task fit impacts smartwatch choice. |
| 44 | Lin, 2014 [57] | 2014 | Mobile Nursing Information System Utilization: The Task-Technology Fit Perspective | CIN: Computers, Informatics, Nursing | Technology-individual fit and organizational readiness significantly influence MNIS usage; organizational support plays a crucial role in adoption. |
| 45 | Yamin et al., 2020 [58] | 2020 | Adoption of telemedicine applications among Saudi citizens during COVID-19 pandemic: An alternative health delivery system | Journal of Infection and Public Health | Task-technology fit and facilitating conditions are crucial for the adoption of telemedicine apps based on wireless sensor networks; R² explains 79.5% of variance in adoption behavior. |
| 46 | Muhammad Shahbaz et al., 2021 [59] | 2021 | Environmental air pollution management system: Predicting user adoption behavior of big data analytics | Technology in Society | Task-technology fit, along with UTAUT factors, strongly influences the adoption of the BDA-EAP management system. Resistance to change negatively impacts adoption, while extrinsic motivation positively moderates adoption. |
| 47 | Hsiao and Chen, 2012 [60] | 2012 | An Investigation on Task-Technology Fit of Mobile Nursing Information Systems for Nursing Performance | CIN: Computers, Informatics, Nursing | Task-technology fit (TTF) positively influences nursing performance through factors such as information identification, acquisition, integration, and interpretation. |
| 48 | Kang et al., 2022 [36] | 2022 | The Acceptance Behavior of Smart Home Health Care Services in South Korea: An Integrated Model of UTAUT and TTF | International Journal of Environmental Research and Public Health | Performance expectancy, effort expectancy, social influence, and task–technology fit significantly influence the behavioral intention and adoption of SHHS. Task characteristics did not affect task–technology fit. |
| 49 | Al-Rahmi et al., 2022 [61] | 2022 | Integrating the Role of UTAUT and TTF Model to Evaluate Social Media Use for Teaching and Learning in Higher Education | Frontiers in Public Health | Task-Technology Fit (TTF) and Behavioral Intention (BI) significantly impact Academic Performance (AP). PEX, EEX, Social Characteristics, and Technology Characteristics influence TTF and BI. Strong interrelationships among UTAUT and TTF constructs improve student performance via social media use. |
| 50 | Hsieh and Lin, 2020 [45] | 2020 | Understanding the performance impact of the epidemic prevention cloud: an integrative model of the task-technology fit and status quo bias | Behaviour & Information Technology | Task and technology characteristics positively influence TTF. TTF enhances utilisation and performance while reducing resistance to use. Resistance is influenced by uncertainty costs (positive), perceived value (negative), but not sunk costs. Resistance negatively affects utilisation but not performance. Utilisation and TTF significantly affect performance. |

**Table S2.** Study characteristics.

| **No** | **Study Population** | | **Study Design** | **Country** |
| --- | --- | --- | --- | --- |
|  | **Sample Size** | **Demographic Variables** |  |  |
| 1 | 274 | Age, Gender, Education, Chronic Disease, Own Mobile Phone, Mobile Phone Usage Experience | Survey Study | Bangladesh |
| 2 | 127 | Age, Gender, Specialisation | Survey Study | Germany |
| 3 | 407 | Age, Gender, Education, Occupation | Quantitative, Cross-sectional | Iran |
| 4 | 746 | Age, Gender, Education, Residence, Diabetic Type, Disease Duration | Quantitative, Cross-sectional | China |
| 5 | 350 | Age, Gender, Education, Marital status, Distance to oncologists | Quantitative, Cross-sectional | Germany |
| 6 | 245 | Age, Gender, Education, Mobile Phone Usage | Quantitative, Cross-sectional | Bangladesh |
| 7 | 413 | Age, Gender, Diabetic Type, Duration of App use | Mixed-methods | Austria, Germany |
| 8 | 707 | Age, Gender, Education | Survey Study | Germany |
| 9 | 58 | Age, Gender, Experience | Cross-sectional Survey | Iran |
| 10 | 127 | Age, Gender, | Mixed methods study | Netherlands |
| 11 | 249 | Age, Gender, Experience, nationality | Cross-sectional Survey | Saudi Arabia |
| 12 | 222 | Age, Education, Income | Cross-sectional Survey | Indonesia |
| 13 | 120 | Age, Gender, Education | Mixed Methods | Portugal |
| 14 | 421 | Age, Gender, | Cross-sectional Survey | Norway |
| 15 | 883 | Age, Gender, Education, Marital status, Occupation, Income, Residence, Religion | Cross-sectional study | Ethiopia |
| 16 | 271 | Age, Gender, Education, Occupation | Quantitative Study using SEM (Structural Equation Modeling) | Saudi Arabia |
| 17 | 7320 | Age, Gender, Application Segment | Empirical study | Germany |
| 18 | 90 | Age, Gender, Occupation | Cross-sectional surveys | Australia |
| 19 | 510 | Age, Gender, Education, Income, Residence | Multistage PLS-SEM | India |
| 20 | 413 | Age, Gender, Stage of Disease, Disease duration | Cross-sectional validation study | Austria, Germany |
| 21 | 257 | Age, Gender, Education | Quantitative Survey Research | Bangladesh |
| 22 | 578 | Age, Gender, Education | Research paper using Structural Equation Modeling (SEM) | India |
| 23 | 568 | Age, Gender, Experience | Survey | India |
| 24 | 118 | Age, Gender, | Quantitative | Indonesia |
| 25 | 152 | Age, Gender, Familiarity with Telemedicine | Quantitative study | UAE |
| 26 | 371 | Age, Gender, | Mixed-Method Study | China |
| 27 | 267 | Age, Gender, | Partial Least Approach - Structural Equation Modeling (PLS-SEM) | France |
| 28 | 710 | Age, Gender, Income | Quantitative study | Germany, USA |
| 29 | 352 | Age, Gender, | Cross-sectional survey | Ghana |
| 30 | 1081 | Age, Gender, | Observational Cross-sectional Study | Portugal |
| 31 | 98 | Age, Gender, Experience, work role, Organization type | Survey | India |
| 32 | 720 | Age, Gender, Experience, Usage Frequency | Survey | Malaysia |
| 33 | 96 | Age, Gender, Occupation | Survey-based quantitative study | Indonesia |
| 34 | 306 | Age, Gender, Education, duration of App use | Original Research | Spain |
| 35 | 1453 | Age, Gender, Education | Empirical study using SEM | China |
| 36 | 406 | Gender, Age, Education, Occupation, HWD usage experience | Cross-sectional survey design | China |
| 37 | 423 | Age , Gender, Education Level | Cross-sectional survey design | Taiwan |
| 38 | 164 | Age, Gender, Occupation | Cross-sectional survey | Palestine (Gaza Strip) |
| 39 | 143 | Gender, Age, Teaching Experience | Descriptive-analytical, cross-sectional survey | Iran (Ahwaz University of Medical Sciences) |
| 40 | 102 | Age, Gender, Professional role | Quasi-experimental posttest-only | Canada |
| 41 | 309 | Age, Gender, Nationality | Cross-sectional survey design | Saudi Arabia |
| 42 | 137 | Gender, Age, Education Level | Cross-sectional survey | Indonesia |
| 43 | 248 | Gender, Age, Occupation, Nationality | Cross-sectional survey design | Qatar |
| 44 | 144 | Gender, Education, Work Experience | Cross-sectional survey | Taiwan |
| 45 | 348 | Age, Gender | Cross-sectional survey | Saudi Arabia |
| 46 | 412 | Gender, Age, Education Level | Cross-sectional survey | Pakistan |
| 47 | 219 | Gender, Age, Work Experience | Cross-sectional survey design | Taiwan |
| 48 | 487 | Gender, Age, Marital Status | Cross-sectional survey | South Korea |
| 49 | 383 | Gender, Age, Discipline (Engineering, Science, Management, Social Sciences) | Survey-based SEM (SmartPLS 3.3.3) | Malaysia |
| 50 | 116 | Gender, Age, Education, Position, Work Experience | Cross-sectional field survey using SmartPLS | Taiwan |

**Table S3.** Study characteristics and UTAUT/TTF path coefficients.

| **#** | **Primary objective** | **Main Theory** | **Dependent Variables** | **Independent Variables** | **Moderators/Mediators** | **Methodology** | **Analysis Techniques** | **Effect Strength (β)** |
| --- | --- | --- | --- | --- | --- | --- | --- | --- |
| 1 | The aim of this study was to develop a theoretical model based on the Unified Theory ofAcceptance and Use of Technology (UTAUT) and then empirically test it for determining the key factorsinfluencing elderly users’ intention to adopt and use the mHealth services. | UTAUT | Behavioral Intention Usage Behaviour | Performance expectancy, Effort expectancy, Social influence, Facilitating Conditions, Technology Anxiety, Resistence to Change | NA | Structured Questionnaire Survey | Partial Least Squares (PLS) - SEM | PE_UI: 0.3193* EE_UI: 0.1879* SI_UI: 0.1441* FC_UI: 0.0298* FC_UB: 0.0979* UI_UB: 0.4148* |
| 2 | To identify supporting and constraining factors that influence outpatient physicians’ acceptance of telemedicine | UTAUT | Intention to use | Performance expectancy, Effort expectancy, Social influence, IT anxiety, Data security | NA | PLS-SEM | Path coefficients, Bootstrapping | PE_UI: 0.397*** EE_UI: 0.134* SI_UI: 0.337*** |
| 3 | Analyze factors influencing telemedicine adoption | UTAUT2 | Intention to use telemedicine | Performance Expectancy, Effort Expectancy, Social Influence, Facilitating Conditions, Hedonic Motivation, Perceived Product Advantage, Perceived Security | Innovativeness, Gender | Survey, Path analysis | Descriptive statistics, Kolmogorov-Smirnov, Path analysis | PE_UI: 0.374*** EE_UI: 0.247*** FC_UI: 0.158*** FC_UB: 0.088*** |
| 4 | Identify determinants of intention to use diabetes management apps | UTAUT | Behavioral Intention | Performance Expectancy, Effort Expectancy, Social Influence, Facilitating Conditions, Perceived Disease Threat, Perceived Privacy Risk | Social Influence, Effort Expectancy, Facilitating Conditions (as mediators for Performance Expectancy) | Survey, Structural Equation Modeling | Descriptive statistics, Structural Equation Modeling | PE_UI: 0.482* EE_UI: 0.482* SI_UI: 0.223* FC_UI: 0.17* FC_UB: 0.073* UI_UB: -0.073* |
| 5 | Assess acceptance of video consultations in cancer care | UTAUT | Behavioral Intention | Age, Gender, Stage of Disease, Digital Confidence, Internet Anxiety, Digital Overload, eHealth Literacy, Personal Trust, Internet Use, Performance Expectancy, Effort Expectancy, Social Influence | None | Survey, Multiple Hierarchical Regression Analysis | Descriptive statistics, Multiple Hierarchical Regression Analysis | PE_UI: 0.24*** EE_UI: 0.26*** SI_UI: 0.34*** |
| 6 | Identify factors influencing elderly’s intention to use m-health services | UTAUT | Behavioral Intention | Performance Expectancy, Effort Expectancy, Social Influence, Facilitating Conditions, Perceived Credibility | None | Survey, Structural Equation Modeling | Descriptive statistics, Structural Equation Modeling | PE_UI: 0.359* EE_UI: 0.197* SI_UI: 0.185* FC_UI: -0.016 FC_UB: 0.082 UI_UB: 0.426* |
| 7 | Predict mHealth acceptance using UTAUT2 model | UTAUT2 | Behavioral Intention | Performance Expectancy, Effort Expectancy, Social Influence, Facilitating Conditions, Hedonic Motivation, Price Value, Habit, Perceived Disease Threat, Trust | None | Mixed-methods, Survey, PLS-SEM | Descriptive statistics, PLS-SEM | PE_UI: 0.285* EE_UI: 0.076 SI_UI: -0.025 FC_UI: 0.071 FC_UB: -0.110* UI_UB: 0.105 |
| 8 | To compare factors influencing the acceptance of lifestyle and therapy apps | UTAUT2 | Behavioral intention | Hedonic motivation, habit, social influence, trust | None specified | Online questionnaire, PLS-SEM | Bootstrapping, correlation analysis | PE_UI: 0.11* EE_UI: -0.056 SI_UI: 0.185*** FC_UI: 0.019 |
| 9 | Identify factors affecting the acceptance of mobile health technology by hematologists | UTAUT | Behavioral Intention | Performance Expectancy, Effort Expectancy, Social Influence, Facilitating Conditions, Usability, Knowledge of IT, Reliability | None specified | Questionnaire, Structural Equation Modeling | Descriptive statistics, PLS-SEM | PE_UI: 0.567* EE_UI: 0.548* SI_UI: 0.743*** FC_UI: 0.548 FC_UB: 0.661* UI_UB: 0.427*** |
| 10 | Investigate key acceptance factors of virtual assistants | UTAUT | Behavioral Intention | Performance Expectancy, Effort Expectancy, Social Influence, Trust | Self-efficacy, Resistance to Change | Mixed methods (qualitative interviews and quantitative survey) | Structural Equation Modeling, Multigroup Analysi | PE_UI: 0.399*** EE_UI: 0.258** SI_UI: 0.114* FC_UI: 0.05 FC_UB: 0.561 UI_UB: 0.531 |
| 11 | Measure the role of trust and information quality in using digital healthcare platforms | UTAUT | Behavioral Intention | Performance Expectancy, Effort Expectancy, Social Influence, Facilitating Conditions, Information Quality, Trust | Experience, Age | Online structured questionnaire | Structural Equation Modeling | PE_UI: 0.194 EE_UI: 0.04 SI_UI: 0.096 FC_UI: 0.233* FC_UB: 0.009 UI_UB: 0.324 |
| 12 | Determine the factors influencing the intention to participate in value co-creation and use iPosyandu | UTAUT | Intention to use iPosyandu, Intention to participate in value co-creation | Performance Expectancy, Effort Expectancy, Social Influence, Facilitating Conditions, Perceived Policy Support, Personal Traits | None specified | PLS-SEM | Structural Equation Modeling | PE_UI: 0.034 EE_UI: 0.623*** SI_UI: -0.009 FC_UI: 0.054 |
| 13 | Assess mHealth adoption factors using UTAUT2 | UTAUT2 | mHealth adoption | Performance Expectancy, Effort Expectancy, Social Influence, Facilitating Conditions, Hedonic Motivation, Price Value, Habit | Age, Gender, Education | Survey | PLS-SEM, fsQCA | PE_UI: 0.45*** EE_UI: 0.08 SI_UI: 0.05 FC_UI: -0.01 |
| 14 | Explore configurations leading to positive and negative impacts of digital technologies | Task-Technology Fit Theory | Work performance | Task characteristics, technology characteristics, individual use context | None | Survey, fsQCA (fuzzy set qualitative comparative analysis) | fsQCA, statistical analysis | PE_UI: 0.348*** EE_UI: 0.312*** SI_UI: 0.057 FC_UI: 0.184*** |
| 15 | To assess DM patients’ intentions to use wearable health devices and its predictors in Ethiopia | UTAUT2 model | Intention to use wearable health devices | Performance expectancy, Effort expectancy, Social influence, Facilitating conditions, Hedonic motivation, Price value, Habit | Age, Gender | Structural equation modeling | Descriptive statistics, Confirmatory factor analysis, Structural equation modeling | PE_UI: 0.306** EE_UI: 0.543** SI_UI: 0.11 FC_UI: 0.131* UI_UB: -0.049 |
| 16 | Evaluate determinants of mIoT adoption among hospital staff in Saudi Arabia | UTAUT-HS | Behavioural Intention to Adopt mIoT | Computer and English language Self-Efficacy (CESE), Performance Expectancy (PE), Social Influence (SI), Perceived Threat to Autonomy (PTA), Confidentiality Concerns (CC) | Age, gender, occupation, education | Quantitative Study using SEM | Structural Equation Modeling (SEM) | PE_UI: 0.148* EE_UI: 0.057 SI_UI: 0.175* FC_UI: 0.002 |
| 17 | Investigate influence factors in an acceptance model on behavioral intention and use behavior for products containing AI in everyday life environment | UTAUT2 | Behavioral intention, Use behavior | Health, Convenience comfort, Sustainability, Safety security, Personal innovativeness, Performance expectancy, Effort expectancy, Social influence, Price value, Hedonic motivation, Habit | None | PLS-Analysis | Structural equation modeling | PE_UI: 0.252* EE_UI: 0.099 SI_UI: 0.146* UI_UB: 0.259* |
| 18 | Investigate allied health professionals’ perspectives pre- and post-implementation of an electronic medical record | Unified Theory of Acceptance and Use of Technology (UTAUT) | User acceptance (Behavioral Intention, Use Behavior) | Performance Expectancy (PE), Effort Expectancy (EE), Social Influence (SI), Facilitating Conditions (FC), Anxiety (AN) | None specified | Cross-sectional surveys with descriptive analysis, Mann–Whitney U tests, PLS-SEM, content analysis | Descriptive analysis, Mann–Whitney U tests, Partial Least Squares Structural Equation Modelling (PLS-SEM), Content Analysis | PE_UI: -0.023 EE_UI: -0.28 SI_UI: 0.058 FC_UI: -0.153 |
| 19 | To quantify the dynamic factors affecting users’ adoption of 5G in emerging countries. | Technology Acceptance Model (TAM) and Unified Theory of Acceptance and Use of Technology (UTAUT) | Behavioral Intention (BI) | Performance Expectancy (PE), Effort Expectancy (EE), Social Factors (SF), Facilitating Factors (FF), Hedonic Motivation (HM), Perceived Benefits (PB), Price Value (PV), Habit (HB), Perceived Trust (PT) | Perceived Trust (PT) | Partial Least Squares Structural Equation Modeling (PLS-SEM) | Structural Equation Modeling (SEM) | PE_UI: 0.352 EE_UI: 0.102 SI_UI: -0.055 FC_UI: 0.109 |
| 20 | To evaluate the extended UTAUT2 model for predicting mHealth acceptance using mobile diabetes applications. | UTAUT2 | Behavioral intention to use mHealth applications | Performance expectancy, Effort expectancy, Social influence, Facilitating conditions, Hedonic motivation, Price value, Habit, Perceived disease threat, Trust | Age, Gender, Experience | Structural equation modeling | Partial least squares (PLS-SEM) | PE_UI: 0.285*** EE_UI: 0.076 SI_UI: -0.025 FC_UI: 0.071 FC_UB: -0.11 UI_UB: 0.105 |
| 21 | Investigate the role of situational constraint and health consciousness in mHealth adoption | UTAUT | Behavioral Intention (BI), Use Behavior (UB) | PE, EE, SI, FC, SC, HC | Situational Constraint, Health Consciousness | Partial Least Squares (PLS) SEM | SmartPLS 3.0 and SPSS 23.0 | PE_UI: 0.152** EE_UI: -0.029** SI_UI: 0.137** FC_UI: 0.194** UI_UB: 0.504** |
| 22 | To explore factors influencing patients' intention towards e-health consultation | Extended UTAUT model | Behavioral intention (BI) | Performance expectancy (PE), Effort expectancy (EE), Social influence (SI), Facilitating condition (FC), Perceived risk (PR), Trust | None mentioned | Survey with SEM | CFA, SEM | PE_UI: 0.229*** EE_UI: 0.247*** SI_UI: 0.078 FC_UI: 0.12*** UI_UB: 0.538*** |
| 23 | Determine impact of UTAUT predictors on intention and usage of EHR and telemedicine | Unified Theory of Acceptance and Use of Technology (UTAUT) | Behavioral Intention, Usage Behavior | Performance Expectancy, Effort Expectancy, Social Influence, Facilitating Conditions | None | Survey | Regression Analysis using AMOS | PE_UI: 0.652*** EE_UI: 0.214*** SI_UI: 0.083*** FC_UB: 0.538*** UI_UB: -0.269*** |
| 24 | To examine the simultaneous effects of performance expectancy, effort expectancy, social influence, facilitating conditions, doctors’ opinion and computer anxiety on the intention to use Telehealth | UTAUT Model | Behavioral Intention | Performance Expectancy, Effort Expectancy, Social Influence, Facilitating Conditions, Doctor's Opinions, Computer Anxiety | None | Survey, PLS-SEM | Path-Model Analysis | PE_UI: 0.24** EE_UI: 0.286** SI_UI: 0.116 FC_UI: 0.271** |
| 25 | To explore factors affecting telemedicine adoption by integrating UTAUT2 and IS Success Model | UTAUT2 and IS Success Model | Telemedicine adoption | Performance expectancy, effort expectancy, social influence, facilitating conditions, hedonic motivation, perceived security, information quality, system quality, service quality, user satisfaction | Removed price value and habit from UTAUT2 | Quantitative, SEM-ANN approach | PLS-SEM and ANN | PE_UI: 0.181* EE_UI: -0.114 SI_UI: -0.088 FC_UI: 0.08 |
| 26 | Examine factors influencing the use intention of mHealth applications based on UTAUT-2 model | UTAUT-2 | Use intention | Performance expectancy, effort expectancy, social influence, facilitating conditions, perceived trust, perceived risk, price value | Gender, Age | Structural Equation Model (SEM), Questionnaires, In-depth Interviews | Descriptive statistics, Structural Equation Modeling (SEM) | PE_UI: 0.4*** EE_UI: 0.4*** SI_UI: 0.14* FC_UI: 0.15*** FC_UB: 0.01 UI_UB: 0.48*** |
| 27 | Examine factors influencing patients’ adoption of the IoT for eHealth | UTAUT | Behavioral Intention (BI) | Performance Expectancy (PE), Effort Expectancy (EE), Social Influence (SI), Facilitating Conditions (FC) | Perceived Risk (PR), Perceived Trust (PT) | PLS-SEM | PLS-SEM | PE_UI: 0.076* EE_UI: 0.17* SI_UI: 0.335* FC_UI: 0.309* |
| 28 | To understand patients’ usage intentions of virtual doctor appointments during the COVID-19 pandemic using UTAUT2 model | UTAUT2 | Usage intention of virtual doctor appointments | Performance expectancy, effort expectancy, social influence, facilitating conditions, habit, hedonic motivation, perceived security, perceived product advantage | Age, gender | Survey | SPSS, AMOS | PE_UI: 0.288*** EE_UI: 0.058 SI_UI: 0.004 FC_UI: 0.011 |
| 29 | Examine use behavior of healthcare professionals towards telemedicine | UTAUT | Behavioral Intention, Use Behavior | Performance Expectancy, Effort Expectancy, Facilitating Conditions | Information Quality | Structural Equation Modeling (SEM) | STATA, SPSS | PE_UI: 0.146* EE_UI: 0.884*** FC_UI: 0.248*** FC_UB: 0.378*** UI_UB: 0.537*** |
| 30 | To validate the UTAUT model for evaluating adherence to the STAYAWAY COVID mobile app | UTAUT | Behavioral Intention | Performance expectancy, Effort expectancy, Social influence, Facilitating conditions, App-related privacy concerns, Innovativeness, COVID-19-related stress | None | Observational cross-sectional study | Cronbach’s alpha, CFA, goodness of fit | PE_UI: 0.753*** EE_UI: 0.04 SI_UI: 0.545*** FC_UI: -0.087* |
| 31 | Investigate factors influencing acceptance and use of a mobile-based IT solution for TB treatment monitoring | UTAUT | Behavioral intention (BI) | Performance expectancy (PE), Effort expectancy (EE), Social influence (SI), Facilitating conditions (FC) | Gender, Age, Experience, Organization Type, Work Role | Survey, Partial Least Squares Structural Equation Modeling (PLS-SEM) | Descriptive statistics, PLS-SEM | PE_UI: 0.314** EE_UI: 0.252* SI_UI: 0.205* FC_UI: 0.284** |
| 32 | Investigate factors influencing consumer intentions towards accepting and using health applications | UTAUT2, Privacy Calculus Model | Behavioral Intentions (BI), Use Behavior (UB) | Performance Expectancy (PE), Effort Expectancy (EE), Social Influence (SI), Facilitating Condition (FC), Habit (HB), Hedonic Motivation (HM), Price Value (PV), Trust in SNIC (TS), Privacy Concern (PC), Perceived Risk (PR), Perceived Credibility (PCR) | None | Survey | Structural Equation Modeling (SEM), Partial Least Squares (PLS) | PE_UI: 0.013*** EE_UI: 0.01** SI_UI: 0.004* FC_UB: 0.08** UI_UB : 0.053 *** |
| 33 | Examine the role of performance expectancy, effort expectancy, social influence, and facilitating factors on the actual usage of mobile health applications | Extended UTAUT Model | Actual Usage Behavior | Performance Expectancy, Effort Expectancy, Social Influence, Facilitating Conditions | Behavioral Intention | Structural Equation Modeling – Partial Least Square | Structural Equation Modeling – Partial Least Square | PE_UI: 0.037* EE_UI : 0.000* SI_UI: 0.043* FC_UI: 0.034 UI_UB : 0.000* |
| 34 | To explore the factors included in the model UTAUT2 directly impacting user behavior around VAs | UTAUT2 | Behavioral intention | Performance expectancy, Effort expectancy, Social influence, Facilitating conditions, Hedonic motivation, Price/value, Habit, Perceived privacy risk, Trust, Personal innovativeness | None | Online survey with SEM analysis | SEM Analysis | PE_UI: 0.136 EE_UI: -0.141 SI_UI:-.0.008 FC_UI: 0.17 |
| 35 | Examine factors influencing mHealth continuance use of elders with chronic diseases | ECM-ISC and UTAUT | Continuance intention | Performance expectancy, Effort expectancy, Social influence, Facilitating conditions | None | Questionnaire survey | Structural equation modeling (SEM) | PE_UI: 0.383*** EE_UI: 0.202*** FC_UI: 0.214*** |
| 36 | To develop and empirically test an integrated model of UTAUT and TTF to understand consumer acceptance of healthcare wearable devices (HWDs). | TTF and UTAUT | Behavioral Intention to Use HWDs | Task and Technology characteristics | Task-technology fit | Self-administered questionnaire survey | Partial least squares structural equation modeling (PLS-SEM) | Tech - TTF: 0.726 TC - TTF : 0.118 TTF - UI : 0.219 |
| 37 | To integrate Task-Technology Fit (TTF) and Innovation Diffusion Theory (IDT) to evaluate young users’ intention to use a mobile cloud healthcare system for diabetes preventive care. | TTF | Intention to use the system | Task characteristics, Technology characteristics, Complexity, Observability, Relative Advantage | Task-technology fit | Questionnaire survey | Structural Equation Modeling (SEM) | Tech - TTF: 0.780 TC - TTF : 0.193 TTF - UI : 0.407 |
| 38 | To extend the TTF model by incorporating nurses’ attitudes and evaluate the predictive power of regression and neural network models for nurse satisfaction with HIS. | TTF | Nurses’ satisfaction with HIS | Task characteristics, Technology characteristics, Task-technology fit, Nurses’ attitude | Nurses’ satisfaction (as mediator) | Questionnaire survey with AI prediction models | Artificial Neural Network (ANN), Regression analysis | Tech - TTF: 0.494 TC - TTF : 0.261 TTF - UI : 0.697 |
| 39 | To identify the key determining factors influencing faculty members’ intention to adopt e-learning during COVID-19 by integrating UTAUT and TTF models. | TTF and UTAUT | Intention to adopt e-learning | Technology characteristics, Task characteristics | Task-technology fit | Questionnaire survey | Spearman correlation, Regression analysis | Tech - TTF: 0.652 TC - TTF : 0.525 TTF - UI : 0.244 |
| 40 | To develop and validate a conceptual model exploring how mHealth affects physicians’ perceived quality of care (PQoC) in a hospital setting. | TTF | Perceived Quality of Care (PQoC) | Task characteristics, Technology characteristics, Self-efficacy, TTF, mHealth utilization | TTF, Self-efficacy (as mediators) | Observational survey | Partial Least Squares-Structural Equation Modeling (PLS-SEM) | Tech - TTF: 0.479 TC - TTF : 0.337 |
| 41 | To predict the factors influencing adoption of the Tawakkalna COVID-19 contact-tracing app in Saudi Arabia using TAM, PCT, and TTF, and validate using SEM and ANN. | TTF | Behavioral intention | Perceived ease of use, Perceived usefulness, Task features, Mobility, Privacy risk | Task-technology fit | Questionnaire survey | Structural Equation Modeling (SEM), Artificial Neural Network (ANN) | Tech - TTF: 0.430 TC - TTF : 0.450 TTF - UI : 0.269 |
| 42 | To explore factors affecting the continuance usage intention (CUI) of telemedicine apps in Jakarta using Task-Technology Fit (TTF) theory. | TTF | Continuance Usage Intention (CUI) | Technology characteristics, Task characteristics, Awareness, Self-efficacy | Task-technology fit (TTF) | Online questionnaire survey | Structural Equation Modeling (SEM-PLS) | Tech - TTF: 0.396 TC - TTF : 0.104 TTF - UI : 0.191 |
| 43 | To develop and validate a Task-Technology-Identity Fit (TTIF) model that predicts smartwatch utilisation and satisfaction using both SEM and ANN analysis. | TTF | User Satisfaction | Task-Technology Fit, Technology-Identity Fit, Utilisation | Task-Technology Fit, Technology-Identity Fit as mediators | Questionnaire survey | Structural Equation Modeling (SEM), Artificial Neural Network (ANN) | Tech - TTF: 0.382 TC - TTF : 0.382 TTF - UI : 0.680 |
| 44 | To investigate the effectiveness of mobile nursing information systems (MNIS) using an extended TTF model that includes organizational readiness and separates TTF into task-technology fit (TaTeF) and technology-individual fit (TeIF). | TTF | System Usage | Clinical task characteristics, MNIS characteristics, Computer self-efficacy, Organizational readiness | Task-technology fit, Technology-individual fit | Questionnaire survey | Partial Least Squares Structural Equation Modeling (PLS-SEM) | Tech - TTF: 0.298 TC - TTF : 0.352 TTF - UI : 0.300 |
| 45 | To investigate Saudi citizens’ behavioral intention to adopt wireless sensor network (WSN)-based telemedicine apps during COVID-19, extending UTAUT with TTF. | TTF | Intention to adopt telemedicine apps | Performance expectancy, Social influence, Effort expectancy, Facilitating conditions, Task-technology fit, Awareness, Self-efficacy | Task-technology fit as a moderator | Online questionnaire survey | Structural Equation Modeling (SEM) | Tech - TTF: 0.209 TC - TTF : 0.489 TTF - UI : 0.508 |
| 46 | To propose a big data analytics (BDA) system for environmental air pollution (EAP) management and examine user adoption behavior using TTF and UTAUT frameworks. | TTF and UTAUT | Behavioral Intention | Task characteristics, Technology characteristics | Resistance to change, Extrinsic motivation as moderators | Structured questionnaire survey | Structural Equation Modeling (SEM) | Tech - TTF: 0.267 TC - TTF : 0.284 TTF - UI : 0.140 |
| 47 | To investigate the relationship between nursing task characteristics, m-NIS characteristics, TTF, and nursing performance using a modified TTF model. | TTF | Nursing Performance | Nursing task characteristics, m-NIS characteristics, Information acquisition, integration, and interpretation | Task-technology fit | Questionnaire survey | Structural Equation Modeling (SEM) | Tech - TTF: 0.270 TC - TTF : 0.308 TTF - UI : 0.312 |
| 48 | To analyze the user acceptance behavior of smart home health care services (SHHSs) in South Korea using an integrated UTAUT and TTF model. | TTF | Behavioral Intention, Adoption | Performance expectancy, Effort expectancy, Social influence, Facilitating conditions, Task–Technology Fit | None | Online survey | Partial Least Squares Structural Equation Modeling (PLS-SEM) | Tech - TTF: 0.517 TC - TTF : -0.007 TTF - UI : -0.209 |
| 49 | To integrate UTAUT and TTF models to evaluate social media use in teaching and learning in higher education and assess their impact on academic performance. | TTF and UTAUT | Behavioral Intention, Task-Technology Fit, Academic Performance | Technology Characteristics | None explicitly tested | Structural Equation Modeling integrating UTAUT and TTF | Partial Least Squares Structural Equation Modeling (PLS-SEM), Bootstrapping, Reliability/Validity Testing | Tech - TTF: 0.199 TTF - UI : 0.107 |
| 50 | To examine the performance impact and user resistance behavior toward the epidemic prevention cloud (EPC) using an integrated Task-Technology Fit (TTF) and Status Quo Bias (SQB) model. | TTF | Utilisation, Performance, Resistance to Use | Task Characteristics, Technology Characteristics, TTF, Uncertainty Costs, Perceived Value, Sunk Costs | None explicitly tested | Integrated TTF and Status Quo Bias (SQB) theoretical model | Partial Least Squares Structural Equation Modeling (PLS-SEM), Bootstrapping, Reliability and Validity Testing | Tech - TTF: 0.474 TC - TTF : 0.277 TTF - UI : 0.712 |

**References**

11. Wang H, Tao D, Yu N, Qu X. Understanding consumer acceptance of healthcare wearable devices: An integrated model of UTAUT and TTF. Int J Med Inform. Jul 2020;139:104156. [doi: ] [Medline: 32387819]

12. Candra S, Williar AY, Princes E, Loang OK, Delphin G, Basmantra IN. Using an extended UTAUT theory to examine the consumer behavior of m-health apps: preliminary results. Presented at: 2022 International Conference on ICT for Smart Society (ICISS); Aug 10-11, 2022; Bandung, Indonesia. [doi: ]

15. Duarte P, Pinho JC. A mixed methods UTAUT2-based approach to assess mobile health adoption. J Bus Res. Sep 2019;102:140-150. [doi: ]

16. Thabet Z, Albashtawi S, Ansari H, Al-Emran M, Al-Sharafi MA, AlQudah AA. Exploring the factors affecting telemedicine adoption by integrating UTAUT2 and IS success model: a hybrid SEM–ANN approach. IEEE Trans Eng Manage. 2023;71:8938-8950. [doi: ]

17. Dash A, Sahoo AK. Exploring patient’s intention towards e-health consultation using an extended UTAUT model. JET. Nov 21, 2022;16(4):266-279. [doi: ]

18. Diel S, Doctor E, Reith R, Buck C, Eymann T. Examining supporting and constraining factors of physicians’ acceptance of telemedical online consultations: a survey study. BMC Health Serv Res. Oct 19, 2023;23(1):1128. [doi: ] [Medline: 37858170]

19. Qvist A, Mullan L, Nguyen L, et al. Investigating allied health professionals’ attitudes, perceptions and acceptance of an electronic medical record using the Unified Theory of Acceptance and Use of Technology. Aust HEALTH Rev. Feb 2024;48(1):16-27. [doi: ] [Medline: 38281312]

20. Dadhich M, Hiran KK, Rao SS, Sharma R. Factors influencing patient adoption of the IoT for E-Health Management Systems (e-HMS) using the UTAUT model. IJACI. Jan 2022;13(1):1-18. [doi: ]

21. Alkhalifah A, Bukar UA. Examining the prediction of COVID-19 contact-tracing app adoption using an integrated model and hybrid approach analysis. Front Public Health. 2022;10:847184. [doi: ] [Medline: 35685757]

22. Tian XF, Wu RZ. Determinants of the mobile health continuance intention of elders with chronic diseases: an integrated framework of ECM-ISC and UTAUT. Int J Environ Res Public Health. Aug 12, 2022;19(16):9980. [doi: ] [Medline: 36011615]

23. Schomakers EM, Lidynia C, Vervier LS, Calero Valdez A, Ziefle M. Applying an extended UTAUT2 model to explain user acceptance of lifestyle and therapy mobile health apps: survey study. JMIR Mhealth Uhealth. Jan 18, 2022;10(1):e27095. [doi: ] [Medline: 35040801]

24. Arfi WB, Nasr IB, Kondrateva G, Hikkerova L. The role of trust in intention to use the IoT in eHealth: application of the modified UTAUT in a consumer context. Technol Forecast Soc Change. Jun 2021;167:120688. [doi: ]

25. Venugopal S. An analysis of the impact of UTAUT predictors on the intention and usage of electronic health records and telemedicine from the perspective of clinical staffs. IJOMAM. Nov 30, 2018;1(4):263-269. URL: <http://ijomam.com/ijomam-issue-4> [doi: ]

26. Farhady S, Sepehri MM, Pourfathollah AA. Evaluation of effective factors in the acceptance of mobile health technology using the Unified Theory of Acceptance and Use of Technology (UTAUT), case study: blood transfusion complications in thalassemia patients. Med J Islam Repub Iran. 2020;34(1):1-7. [doi: ] [Medline: 33306059]

27. Hoque R, Sorwar G. Understanding factors influencing the adoption of mHealth by the elderly: an extension of the UTAUT model. Int J Med Inform. May 2017;101:75-84. [doi: ] [Medline: 28347450]

28. Lathifah A, Putro US, Rinawan FR, Novani S, Hasyimi V, Tiara AR. Understanding participation in value co-creation and acceptance of iPosyandu by extending UTAUT among community health workers. CommIT. 2023;17(2):185-197. [doi: ]

29. Schmitz A, Díaz-Martín AM, Yagüe Guillén MJ. Modifying UTAUT2 for a cross-country comparison of telemedicine adoption. Comput Human Behav. May 2022;130:107183. [doi: ] [Medline: 35017788]

30. Napitupulu D, Yacub R, Putra A. Factor influencing of telehealth acceptance during COVID-19 outbreak: extending UTAUT model. IJIES. 2021;14(3):267-281. [doi: ]

31. García de Blanes Sebastián M, Sarmiento Guede JR, Antonovica A. Application and extension of the UTAUT2 model for determining behavioral intention factors in use of the artificial intelligence virtual assistants. Front Psychol. 2022;13:993935. [doi: ] [Medline: 36329748]

32. Nurtsch A, Teufel M, Jahre LM, et al. Drivers and barriers of patients’ acceptance of video consultation in cancer care. Digit Health. 2024;10:20552076231222108. [doi: ] [Medline: 38188860]

33. Alharbi F. The use of digital healthcare platforms during the COVID-19 pandemic: the consumer perspective. Acta Inform Med. Mar 2021;29(1):51-58. [doi: ] [Medline: 34012214]

34. Araújo I, Grilo A, Silva C. Portuguese validation of the Unified Theory of Acceptance and Use of Technology Scale (UTAUT) to a COVID-19 mobile application: a pilot study. Healthcare (Basel). Jul 3, 2023;11(13):1916. [doi: ] [Medline: 37444750]

35. Owusu Kwateng K, Darko-Larbi O, Amanor K. A modified UTAUT2 for the study of telemedicine adoption. Int J Healthc Manag. Apr 3, 2023;16(2):207-223. [doi: ]

36. Kang HJ, Han J, Kwon GH. The acceptance behavior of smart home health care services in South Korea: an integrated model of UTAUT and TTF. Int J Environ Res Public Health. Oct 14, 2022;19(20):13279. [doi: ] [Medline: 36293859]

37. Walle AD, Jemere AT, Tilahun B, et al. Intention to use wearable health devices and its predictors among diabetes mellitus patients in Amhara region referral hospitals, Ethiopia: using modified UTAUT-2 model. Inform Med Unlocked. 2023;36:101157. [doi: ]

38. Nadaf M, Mousavi SJ. Using UTAUT2 model for explaining telemedicine adoption, evidence from Iran. IOH. 2022;19(1):538-554. [doi: ]

39. Alomari A, Soh B. Determinants of medical internet of things adoption in healthcare and the role of demographic factors incorporating modified UTAUT. IJACSA. 2023;14(7):17-31. [doi: ]

40. Gansser OA, Reich CS. A new acceptance model for artificial intelligence with extensions to UTAUT2: an empirical study in three segments of application. Technol Soc. May 2021;65:101535. [doi: ]

41. Zhang Y, Liu C, Luo S, et al. Factors influencing patients’ intentions to use diabetes management apps based on an extended Unified Theory of Acceptance and Use of Technology model: web-based survey. J Med Internet Res. Aug 13, 2019;21(8):e15023. [doi: ] [Medline: 31411146]

42. Quaosar G, Hoque MR, Bao Y. Investigating factors affecting elderly’s intention to use m-Health services: an empirical study. Telemed J E Health. Apr 2018;24(4):309-314. [doi: ] [Medline: 28976824]

43. Bile Hassan I, Murad MAA, El-Shekeil I, Liu J. Extending the UTAUT2 model with a privacy calculus model to enhance the adoption of a health information application in Malaysia. Informatics (MDPI). 2022;9(2):31. [doi: ]

44. Seethamraju R, Diatha KS, Garg S. Intention to use a mobile-based information technology solution for tuberculosis treatment monitoring – applying a UTAUT model. Inf Syst Front. Feb 2018;20(1):163-181. [doi: ]

45. Hsieh PJ, Lin WS. Understanding the performance impact of the epidemic prevention cloud: an integrative model of the task-technology fit and status quo bias. Behav Inf Technol. Aug 2, 2020;39(8):899-916. [doi: ]

46. Schretzlmaier P, Hecker A, Ammenwerth E. Predicting mHealth acceptance using the UTAUT2 technology acceptance model: a mixed-methods approach. Stud Health Technol Inform. May 2, 2023;301:26-32. [doi: ] [Medline: 37172148]

47. van Bussel MJP, Odekerken-Schröder GJ, Ou C, Swart RR, Jacobs MJG. Analyzing the determinants to accept a virtual assistant and use cases among cancer patients: a mixed methods study. BMC Health Serv Res. Jul 9, 2022;22(1):890. [doi: ] [Medline: 35804356]

48. Baum U, Kühn F, Lichters M, et al. Neurological outpatients prefer EEG home-monitoring over inpatient monitoring-an analysis based on the UTAUT model. Int J Environ Res Public Health. Oct 13, 2022;19(20):13202. [doi: ] [Medline: 36293783]

49. Barua Z, Barua A. Acceptance and usage of mHealth technologies amid COVID-19 pandemic in a developing country: the UTAUT combined with situational constraint and health consciousness. JET. Jun 1, 2021;15(1):1-22. [doi: ]

50. Zhu Y, Zhao Z, Guo J, et al. Understanding use intention of mHealth applications based on the Unified Theory of Acceptance and Use of Technology 2 (UTAUT-2) model in China. Int J Environ Res Public Health. Feb 10, 2023;20(4):3139. [doi: ] [Medline: 36833830]

51. Wang SL, Lin HI. Integrating TTF and IDT to evaluate user intention of big data analytics in mobile cloud healthcare system. Behav Inf Technol. Sep 2, 2019;38(9):974-985. [doi: ]

52. Alhendawi KM. Task-technology fit model: modelling and assessing the nurses’ satisfaction with health information system using AI prediction models. Int J Healthc Manag. Jan 2, 2024;17(1):12-24. [doi: ]

53. Abdekhoda M, Dehnad A, Zarei J. Factors influencing adoption of e-learning in healthcare: integration of UTAUT and TTF model. BMC Med Inform Decis Mak. Dec 9, 2022;22(1):327. [doi: ] [Medline: 36494800]

54. O’Connor Y, Andreev P, O’Reilly P. MHealth and perceived quality of care delivery: a conceptual model and validation. BMC Med Inform Decis Mak. Feb 27, 2020;20(1):41. [doi: ] [Medline: 32103746]

55. Wijaya L, Ng KC, Sihombing PR. Assessing determinants of the telemedicine applications continuance usage intention with TTF theory. Presented at: 2023 8th International Conference on Business and Industrial Research (ICBIR); May 18-19, 2025; Bangkok, Thailand. [doi: ]

56. El-Masri M, Al-Yafi K, Kamal MM. A task-technology-identity fit model of smartwatch utilisation and user satisfaction: a hybrid SEM-neural network approach. Inf Syst Front. 2023;25(2):835-852. [doi: ] [Medline: 35378907]

57. Lin TC. Mobile nursing information system utilization: the task-technology fit perspective. Comput Inform Nurs. Mar 2014;32(3):129-137. [doi: ] [Medline: 24419090]

58. Yamin MAY, Alyoubi BA. Adoption of telemedicine applications among Saudi citizens during COVID-19 pandemic: an alternative health delivery system. J Infect Public Health. Dec 2020;13(12):1845-1855. [doi: ] [Medline: 33172819]

59. Shahbaz M, Gao C, Zhai L, Shahzad F, Khan I. Environmental air pollution management system: predicting user adoption behavior of big data analytics. Technol Soc. Feb 2021;64:101473. [doi: ]

60. Hsiao JL, Chen RF. An investigation on task-technology fit of mobile nursing information systems for nursing performance. Comput Inform Nurs. May 2012;30(5):265-273. [doi: ] [Medline: 22156768]

61. Schretzlmaier P, Hecker A, Ammenwerth E. Extension of the Unified Theory of Acceptance and Use of Technology 2 model for predicting mHealth acceptance using diabetes as an example: a cross-sectional validation study. BMJ Health Care Inform. Nov 2022;29(1):e100640. [doi: ] [Medline: 36379608]

62. Wu P, Zhang RT, Zhu XM, Liu ML. Factors influencing continued usage behavior on mobile health applications. Healthcare (Basel). Jan 21, 2022;10(2):2. [doi: ] [Medline: 35206823]
