# Supplementary material for: Determinants of Health Care Technology Adoption Using an Integrated Unified Theory of Acceptance and Use of Technology and Task Technology Fit Model: Systematic Review and Meta-Analysis
Source: J Med Internet Res. 2025 Dec 30;27:e64524. doi: 10.2196/64524 (PMC12753102; doi:10.2196/64524)
Supplement: Multimedia Appendix 2 [file jmir-v27-e64524-s002.docx]

**Search Terms and Strategy- Ovid MEDLINE and Other Databases**

| 1 | ("Unified Theory of Acceptance and Use of Technology" or "UTAUT") | 777 |
| --- | --- | --- |
| 2 | ("Healthcare Technology" or "Electronic Health Records" or "EHR" or "Telemedicine" or "Mobile Health" or "mHealth" or "Health Information Technology" or "HIT" or "Wearable Devices" or "Artificial Intelligence" or "AI") | 277211 |
| 3 | ("Adoption" or "Acceptance" or "Intention to Use" or "Usage Behavior") | 237817 |
| 4 | ("Survey" or "Cross-sectional" or "Quantitative" or "Empirical Study" or "Structural Equation Modeling" or "SEM" or "Meta-analysis") | 2584987 |
| 5 | 1 and 2 and 3 and 4 | 178 |
| 6 | limit 5 to (english language and yr="2012 - 2025") | 168 |

| **Database** | **Search Syntax** |
| --- | --- |
| **Medline** | (healthcare OR "health care" OR clinic* OR hospital* OR patient* OR "digital health" OR ehealth OR mhealth OR telehealth OR telemedicine OR "electronic health record*" OR EHR OR EMR OR "health information technology") AND ("unified theory of acceptance and use of technology" OR UTAUT OR "technology acceptance") OR ("task-technology fit" OR TTF) AND (adopt* OR accept* OR "intention to use" OR "behavioral intention" OR usage OR utilisation OR utilization) AND English[lang] AND yr="2012-Current" |
| **Embase** | (healthcare OR "health care" OR clinic* OR hospital* OR patient* OR "digital health" OR ehealth OR mhealth OR telehealth OR telemedicine OR "electronic health record*" OR EHR OR EMR OR "health information technology") AND ('unified theory of acceptance and use of technology' OR UTAUT OR 'technology acceptance') OR ('task technology fit' OR TTF) AND (adopt* OR accept* OR "intention to use" OR "behavioral intention" OR usage OR utilisation OR utilization) AND English[lang] AND [2012-2025] AND human |
| **IEEE Xplore** | (("healthcare" OR "health care" OR hospital* OR clinic* OR patient* OR "digital health" OR ehealth OR mhealth OR telehealth OR telemedicine OR "electronic health record*" OR EHR OR EMR OR "health information technology") AND (technology OR "health information system*")) AND (("unified theory of acceptance and use of technology" OR UTAUT) OR ("task technology fit" OR TTF)) AND (adopt* OR accept* OR "intention to use" OR "behavioral intention" OR usage)  Search result:  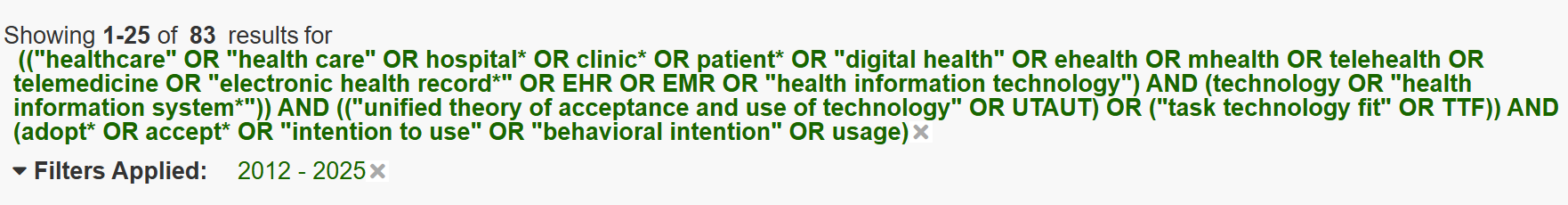 |
| **ScienceDirect** | TITLE-ABS-KEY ( ("healthcare" OR "health care" OR patient* OR hospital* OR clinic* OR "digital health" OR ehealth OR mhealth OR telehealth OR telemedicine OR "electronic health record*" OR EHR OR EMR OR "health information technology") AND ("unified theory of acceptance and use of technology" OR UTAUT OR "task technology fit" OR TTF) AND (adopt* OR accept* OR "intention to use" OR "behavioral intention" OR usage) ) AND PUBYEAR > 2011 AND PUBYEAR < 2026 AND LANGUAGE ( english ) |
| **Scopus** | TITLE-ABS-KEY ( ("healthcare" OR "health care" OR patient* OR hospital* OR clinic* OR "digital health" OR ehealth OR mhealth OR telehealth OR telemedicine OR "electronic health record*" OR EHR OR EMR OR "health information technology") AND ("unified theory of acceptance and use of technology" OR UTAUT OR "task technology fit" OR TTF) AND (adopt* OR accept* OR "intention to use" OR "behavioral intention" OR usage) ) AND ( LIMIT-TO ( LANGUAGE , "English" ) ) AND ( PUBYEAR > 2011 ) AND ( LIMIT-TO ( SUBJAREA , "MEDI" ) OR LIMIT-TO ( SUBJAREA , "COMP" ) ) |
| **CINAHL** | (healthcare OR "health care" OR hospital* OR clinic* OR patient* OR "digital health" OR ehealth OR mhealth OR telehealth OR telemedicine OR "electronic health record*" OR EHR OR EMR OR "health information technology") AND ("unified theory of acceptance and use of technology" OR UTAUT) OR ("task technology fit" OR TTF) AND (adopt* OR accept* OR "intention to use" OR "behavioral intention" OR usage OR utilisation OR utilization) T AND Limiters: English; Publication Date 2012-2025 |
| **Web of Science** | TS=(("healthcare" OR "health care" OR patient* OR hospital* OR clinic* OR "digital health" OR ehealth OR mhealth OR telehealth OR telemedicine OR "electronic health record*" OR EHR OR EMR OR "health information technology") AND ("unified theory of acceptance and use of technology" OR UTAUT OR "task technology fit" OR TTF) AND (adopt* OR accept* OR "intention to use" OR "behavioral intention" OR usage)) AND LA=(English) AND PY=(2012-2025) |
| **Google Scholar** | allintitle: ("unified theory of acceptance and use of technology" OR UTAUT OR "task technology fit" OR TTF) ("healthcare" OR "health care" OR "digital health" OR ehealth OR telehealth OR telemedicine OR "electronic health record*" OR EHR OR EMR OR "health information technology") (adopt* OR accept* OR "intention to use" OR "behavioral intention" OR usage) since 2012 |
