# Supplementary material for: Determinants of Health Care Technology Adoption Using an Integrated Unified Theory of Acceptance and Use of Technology and Task Technology Fit Model: Systematic Review and Meta-Analysis
Source: J Med Internet Res. 2025 Dec 30;27:e64524. doi: 10.2196/64524 (PMC12753102; doi:10.2196/64524)
Supplement: Checklist 1 [file jmir-v27-e64524-s003.docx]

| **Section and Topic** | **Item #** | **Checklist item** | **Location where item is reported** |
| --- | --- | --- | --- |
| **TITLE** | | |  |
| Title | 1 | Identify the report as a systematic review. | Title- Determinants of Healthcare Technology Adoption: A Systematic Review and Meta-Analysis Using an Integrated UTAUT and TTF Model |
| **ABSTRACT** | | |  |
| Abstract | 2 | See the PRISMA 2020 for Abstracts checklist. | Abstract section |
| **INTRODUCTION** | | |  |
| Rationale | 3 | Describe the rationale for the review in the context of existing knowledge. | Introduction Section- 1-3 paragraphs |
| Objectives | 4 | Provide an explicit statement of the objective(s) or question(s) the review addresses. | Introduction- Last Paragraph |
| **METHODS** | | |  |
| Eligibility criteria | 5 | Specify the inclusion and exclusion criteria for the review and how studies were grouped for the syntheses. | Methodology- Study Selection Subsection |
| Information sources | 6 | Specify all databases, registers, websites, organisations, reference lists and other sources searched or consulted to identify studies. Specify the date when each source was last searched or consulted. | Methodology-Study Selection Subsection |
| Search strategy | 7 | Present the full search strategies for all databases, registers and websites, including any filters and limits used. | Available in Multimedia Appendix 3-search strategy used for Medline |
| Selection process | 8 | Specify the methods used to decide whether a study met the inclusion criteria of the review, including how many reviewers screened each record and each report retrieved, whether they worked independently, and if applicable, details of automation tools used in the process. | Methodology-Study Selection Subsection |
| Data collection process | 9 | Specify the methods used to collect data from reports, including how many reviewers collected data from each report, whether they worked independently, any processes for obtaining or confirming data from study investigators, and if applicable, details of automation tools used in the process. | Methodology- Coding Data Subsection |
| Data items | 10a | List and define all outcomes for which data were sought. Specify whether all results that were compatible with each outcome domain in each study were sought (e.g. for all measures, time points, analyses), and if not, the methods used to decide which results to collect. | Methodology-Coding Data Subsection |
|  | 10b | List and define all other variables for which data were sought (e.g. participant and intervention characteristics, funding sources). Describe any assumptions made about any missing or unclear information. | Methodology -Coding Data Subsection |
| Study risk of bias assessment | 11 | Specify the methods used to assess risk of bias in the included studies, including details of the tool(s) used, how many reviewers assessed each study and whether they worked independently, and if applicable, details of automation tools used in the process. | Methodology-study selection-Risk of bias assessment was not conducted. Two reviewers independently assessed the studies to reduce selection bias, with discrepancies resolved by a third reviewer. |
| Effect measures | 12 | Specify for each outcome the effect measure(s) (e.g. risk ratio, mean difference) used in the synthesis or presentation of results. | Methodology-Statistical analysis subsection |
| Synthesis methods | 13a | Describe the processes used to decide which studies were eligible for each synthesis (e.g. tabulating the study intervention characteristics and comparing against the planned groups for each synthesis (item #5)). | Methodology-Statistical analysis subsection |
|  | 13b | Describe any methods required to prepare the data for presentation or synthesis, such as handling of missing summary statistics, or data conversions. | Methodology-Statistical analysis subsection |
|  | 13c | Describe any methods used to tabulate or visually display results of individual studies and syntheses. | Methodology-Statistical analysis subsection |
|  | 13d | Describe any methods used to synthesize results and provide a rationale for the choice(s). If meta-analysis was performed, describe the model(s), method(s) to identify the presence and extent of statistical heterogeneity, and software package(s) used. | Methodology-Statistical analysis subsection |
|  | 13e | Describe any methods used to explore possible causes of heterogeneity among study results (e.g. subgroup analysis, meta-regression). | Methodology-Statistical analysis subsection |
|  | 13f | Describe any sensitivity analyses conducted to assess robustness of the synthesized results. | Methodology-Statistical analysis subsection |
| Reporting bias assessment | 14 | Describe any methods used to assess risk of bias due to missing results in a synthesis (arising from reporting biases). | Methodology-Statistical analysis subsection- Risk of bias due to missing results was not assessed |
| Certainty assessment | 15 | Describe any methods used to assess certainty (or confidence) in the body of evidence for an outcome. | Methodology-Statistical analysis subsection |
| **RESULTS** | | |  |
| Study selection | 16a | Describe the results of the search and selection process, from the number of records identified in the search to the number of studies included in the review, ideally using a flow diagram. | Results- Study selection subsection |
|  | 16b | Cite studies that might appear to meet the inclusion criteria, but which were excluded, and explain why they were excluded. | Results-study selection subsection |
| Study characteristics | 17 | Cite each included study and present its characteristics. | Results-Study characteristics Subsection |
| Risk of bias in studies | 18 | Present assessments of risk of bias for each included study. | Risk of bias assessment was not conducted. Studies were categorized based on their methodological validity. |
| Results of individual studies | 19 | For all outcomes, present, for each study: (a) summary statistics for each group (where appropriate) and (b) an effect estimate and its precision (e.g. confidence/credible interval), ideally using structured tables or plots. | Results-descriptive analysis outcomes subsection |
| Results of syntheses | 20a | For each synthesis, briefly summarise the characteristics and risk of bias among contributing studies. | Risk of bias among contributing studies was not assessed. Characteristics of included studies were summarized by their methodological validity |
|  | 20b | Present results of all statistical syntheses conducted. If meta-analysis was done, present for each the summary estimate and its precision (e.g. confidence/credible interval) and measures of statistical heterogeneity. If comparing groups, describe the direction of the effect. | Results-Meta-Analysis Outcomes subsection |
|  | 20c | Present results of all investigations of possible causes of heterogeneity among study results. | Results-weight analysis outcomes subsection |
|  | 20d | Present results of all sensitivity analyses conducted to assess the robustness of the synthesized results. | Results-weight analysis outcomes subsection |
| Reporting biases | 21 | Present assessments of risk of bias due to missing results (arising from reporting biases) for each synthesis assessed. | Risk of bias due to missing results (arising from reporting biases) was not assessed |
| Certainty of evidence | 22 | Present assessments of certainty (or confidence) in the body of evidence for each outcome assessed. | Results- meta-analysis outcome subsection |
| **DISCUSSION** | | |  |
| Discussion | 23a | Provide a general interpretation of the results in the context of other evidence. | Discussion- 1^st^ Paragraph |
|  | 23b | Discuss any limitations of the evidence included in the review. | Discussion -Limitations and future research directions subsection |
|  | 23c | Discuss any limitations of the review processes used. | Discussion -Limitations and future research directions subsection |
|  | 23d | Discuss implications of the results for practice, policy, and future research. | Discussion -Managerial Implications subsection |
| **OTHER INFORMATION** | | |  |
| Registration and protocol | 24a | Provide registration information for the review, including register name and registration number, or state that the review was not registered. | This systematic review was not registered. |
|  | 24b | Indicate where the review protocol can be accessed, or state that a protocol was not prepared. | A protocol was not prepared for this review |
|  | 24c | Describe and explain any amendments to information provided at registration or in the protocol. | N/A |
| Support | 25 | Describe sources of financial or non-financial support for the review, and the role of the funders or sponsors in the review. | None |
| Competing interests | 26 | Declare any competing interests of review authors. | Conflicts of Interest- None declared |
| Availability of data, code and other materials | 27 | Report which of the following are publicly available and where they can be found: template data collection forms; data extracted from included studies; data used for all analyses; analytic code; any other materials used in the review. | Available in Appendix |

*From:*  Page MJ, McKR²=0.837, MAE=17.47; normal glucose accuracy 95.2%, hyperglycemia 82.6%

IDx-DR* (retinopathy screening): 87.2% sensitivity, 90.7% specificity; EyeArt: 95.5% sensitivity, 85.0% specificity

AUC=0.96 (multimodal) vs. 0.586 (genomics-only)

74.5% accuracy on Chinese Registered Dietitian Exam, 96.43% ketogenic diet responses acceptable/excellent

GPT-4 improved specificity, clarity over GPT-3.5

91% intent recognition, ~4,000 unique users

AMANDA: SUS*=80.625 (usability), MOS*=4.07 (naturalness), 3.98 (accent uniqueness), 3.88 (clarity)

F1 score=0.825 (multilabel ingredient recognition)

98% accuracy, 94% correct answers for diabetes education

Faster insulin titration (15 vs. >56 days, P=0.006), 82.9% adherence

Pending outcomes: ≥5% weight loss, ≥0.2-point A1C reduction

Pending outcomes: HbA1c reduction, improved self-managementenzie JE, Bossuyt PM, Boutron I, Hoffmann TC, Mulrow CD, et al. The PRISMA 2020 statement: an updated guideline for reporting systematic reviews. BMJ 2021;372:n71. doi: 10.1136/bmj.n71
